# Thermal Stability and Decomposition Mechanisms of PVA/PEGDA–PEGMA IPN-Hydrogels: A Multimethod Kinetic Approach

**DOI:** 10.3390/polym17202805

**Published:** 2025-10-21

**Authors:** Akmaral Zh. Sarsenbekova, Ulygbek B. Tuleuov, Akerke T. Kazhmuratova, Abylaikhan N. Bolatbay, Lyazzat Zh. Zhaparova, Yerkeblan M. Tazhbayev

**Affiliations:** Chemistry Faculty, Karaganda Buketov University, Karaganda 100024, Kazakhstan; kazhmuratova@mail.ru (A.T.K.); abylai_bolatbai@mail.ru (A.N.B.); lyazzh@mail.ru (L.Z.Z.); tazhbaev@mail.ru (Y.M.T.)

**Keywords:** hydrogels, interpenetrating networks, thermal stability, decomposition kinetics, activation energy

## Abstract

This paper presents a comprehensive analysis of the thermal stability and decomposition mechanisms of IPN hydrogels based on polyvinyl alcohol (PVA) and a copolymer network of poly(ethylene glycol) diacrylate–poly(ethylene glycol) methacrylate (PEGDA–PEGMA). Using thermogravimetric analysis (TGA/DTG) and multi-approach kinetic analysis (Friedman and Ozawa–Flynn–Wall isoconversion methods, nonparametric kinetics, Shestaka-Berggren model), the influence of composition on the processes of dehydration, thermal destruction, and the distribution of activation energy by degrees of conversion was investigated. The constructed three-dimensional kinetic “landscapes” made it possible to identify characteristic features of the behavior of various samples, including differences in the rate and mechanisms of destruction. It was found that an increase in the content of PVA enhances moisture binding and shifts the T_max_ of dehydration to higher temperatures, while an increase in the concentration of PEGDA forms a denser network that limits moisture retention and accelerates thermal decomposition. Calculation of diffusion coefficients using the Fick model showed a decrease in D with an increase in network density, which reflects an increase in resistance to moisture mass transfer. The combination of the data obtained demonstrates the multistage nature of thermal destruction and allows for the targeted selection of hydrogel compositions for biomedical, environmental, and materials science applications, including drug delivery systems, sorbents and heat-resistant coatings.

## 1. Introduction

Hydrogels are three-dimensional polymer networks with high hydrophilicity and the ability to retain significant amounts of water, which determines their wide application in biomedicine, pharmaceuticals, and materials science [1]. Owing to their adjustable structure and various synthesis methods, they are used as sorbents, drug carriers, wound dressings, and matrices for tissue engineering [2,3]. Interpenetrating polymer networks (IPN hydrogels), which combine chemical and physical cross-linking mechanisms, are of particular interest. This architecture provides higher stability and controllable properties, making these materials promising for both medicine and ecology.

One of the most studied components of hydrogels is polyvinyl alcohol (PVA), which is water-soluble, biocompatible, and highly durable and has film-forming properties. These characteristics determine its use in membrane materials, medical devices, and drug delivery systems. Recent studies have also emphasized the structural and mechanical characteristics of PVA-based hydrogels, confirming their versatility and broad potential for applications in biomedicine and materials science [4]. These works have made a significant contribution to understanding the fundamental properties of PVA hydrogels and have laid the foundation for further research directions.

The results obtained in this study should be considered within the broader context of current advances in the development of functional hydrogels. Yang et al. [5] demonstrated that thermosensitive PNIPA-based hydrogels exhibit high thermal stability and even flame resistance due to the molecular design of their structure. Li et al. [6] highlighted the importance of porous and hierarchical architectures that provide multiscale stability and efficient heat transfer, which are crucial for the durability of complex hydrogel systems. Xin and Lyu [7] summarized various strategies for enhancing thermal stability—from antifreeze additives and nanocomposites to ionogels—thereby expanding the applicability of hydrogels under extreme conditions. Lv et al. [8] focused on the biomedical prospects of thermosensitive hydrogels with programmable degradation profiles and injectable forms.

In contrast to these functionally oriented studies, the present work provides a mechanistic contribution: the systematic application of isoconversional, nonparametric, and autocatalytic approaches has revealed how network density and polymer composition determine the staged decomposition and energetic characteristics of IPN hydrogels. This detailed kinetic characterization complements contemporary research by offering a fundamental understanding of how the stability and controlled degradation of polymer networks can be tuned at the molecular level. In recent years, there has been increased interest in studying the thermal behavior and decomposition kinetics of PVS, as these parameters are directly related to the operational reliability of materials [9,10]. To increase the stability of hydrogels, a chemical cross-linking agent, polyethylene glycol diacrylate (PEGDA), is introduced into their matrix, and an additional polymer network based on polyethylene glycol methacrylate (PEGMA) is formed. This combination allows the density of the spatial structure to be adjusted and the morphology of the hydrogels to be controlled, which directly affects their moisture retention and mechanical characteristics. Polyvinyl alcohol (PVA) contributes to the strengthening of the system by forming hydrogen bonds and creating a denser and more stable network structure. This increases the elasticity and strength of the material and gives it the ability to self-repair.

A number of studies have confirmed the significance of these factors. For example, Tsioptsias et al. [9] showed that physically cross-linked PVS films undergo structural changes due to hydrogen bonds, which affects their thermal stability. Alhulaybi and Dubdub [10] established the multistage nature of PVA pyrolysis and demonstrated the applicability of various isoconversion and model methods to describe the process. Ghorpade et al. [11] demonstrated the controlled release of drugs from PVA/carboxymethylcellulose compositions, and Abad et al. [12] revealed an increase in mechanical strength in radiation-synthesized mixtures of PVP and kappa-carrageenan. Ali and Zaidi [13] established the dependence of swelling on the PVA content in copolymer hydrogels, while Nie et al. [14] emphasized the role of ordered structures in controlling molecule transport. Research on “smart” hydrogels [15,16], as well as new composites and membranes [17,18], has expanded the functional capabilities of materials, but their attention has been focused mainly on biomedical applications and operational stability.

Despite significant progress, some issues remain unresolved. Most studies focus either on individual polymers, such as PVA [9,10] or PEGDA/PEGMA [17,18], or on the general characteristics of multifunctional hydrogels [15,16]. Thermal behavior has often been considered fragmentarily, at the level of individual stages of dehydration or destruction, without comprehensive analysis. There are no systematic studies of IPN hydrogels of PVA/PEGDA–PEGMA with varying compositions in the literature. The comparison of different kinetic approaches has also been insufficiently studied: isoconversion methods have been applied to PVS, but they have rarely been compared with model and nonparametric methods in the analysis of complex IPN systems.

The novelty of this study is that, for the first time, a comprehensive analysis of IPN hydrogels of PVA/PEGDA–PEGMA with varying PVA and PEGDA contents was carried out using isoconversion, model, and nonparametric methods.

The aim of the work is to establish the influence of the composition of hydrogels on the processes of dehydration, thermal decomposition, and energy characteristics that determine the stability and kinetic behavior of these systems.

## 2. Materials and Methods

### 2.1. Materials

Poly(ethylene glycol) diacrylate (PEGDA, average molecular weight *M_n_* = 575 g/mol, purity > 98%) and poly(ethylene glycol) methyl acrylate (PEGMA, *M_n_* = 360 g/mol, purity > 98%) were purchased from Sigma-Aldrich (St. Louis, MO, USA). Polyvinyl alcohol (PVA, average molecular weight 195,000 g/mol, purity > 98%) was purchased from Sigma-Aldrich (St. Louis, MO, USA). Lithium 2,4,6-trimethylbenzoylphosphinate (TPO-Li, purity > 97%) was supplied by Merck (Darmstadt, Germany).

### 2.2. Sample Preparation

**Figure 1:**
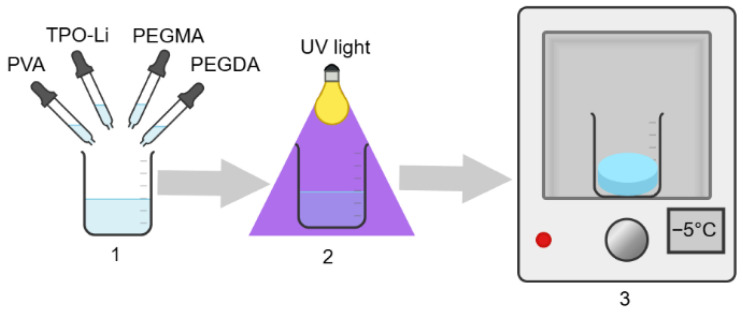
Schematic representation of the synthesis of IPN hydrogels of PVA/PEGDA–PEGMA (PEGMA 11 wt.% in all samples, H_2_O 75–81 wt.%) 1. Mixing of PEGMA monomer, PEGDA crosslinking agent, TPO Li initiator, and PVA solution. 2. Photocrosslinking of the PEGMA/PEGDA network under UV light. 3. Crosslinking of the PVA network with microcrystals using the freeze–thaw method.

The stages of obtaining PVA/PEGMA/PEGDA hydrogels are shown in Figure 1. Dissolution of 9 wt.% PVA in water was performed according to the procedure described earlier [19]. The required amount of PEGMA, PEGDA, and aqueous PVA solution was mixed; the volume was adjusted with Milli-Q water; and then the mixtures were homogenized using a T10 basic ULTRA-TURRAX disperser (IKA, Breisgau, Germany). After that, 0.25 wt.% of the TPO-Li photosensitizer was added to the system, mixed on a magnetic stirrer, and 5 mm thick solutions were exposed to UV irradiation at 365 nm (intensity 9 W) for several minutes.

**Figure 2:**
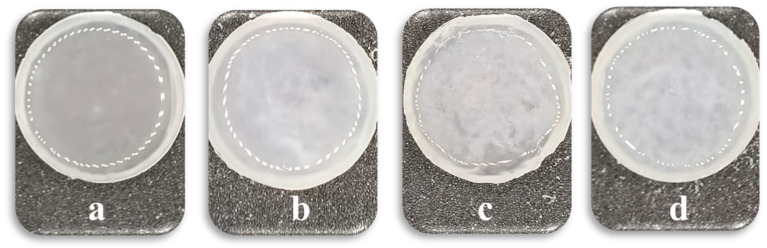
Macrophotographs of IPN hydrogels after freezing/thawing with different PVA contents: (**a**) 1 wt.%, (**b**) 2 wt.%, (**c**) 3 wt.% (with PEGMA fixed at 11 wt.% and PEGDA at 7 wt.%), and (**d**) 3 wt.% (with both PEGMA and PEGDA fixed at 11 wt.%).

After preparation, the hydrogels were frozen at −5 °C for 2 h and then thawed at room temperature for 8 h to form microcrystals in the PVA network (Figure 1 and Figure 2). The frequency dependencies of the storage and loss moduli (G′ and G″) are presented in the Supplementary Materials S1 and S2 (Figures S1 and S2).

### 2.3. Procedure for Studying the Thermal Behavior and Kinetics of Hydrogels

The thermal behavior of PVA/PEGDA–PEGMA-based hydrogels (PEGMA 11 wt.% in all samples, H_2_O 75–81 wt.%) was studied using a Labsys Evo TG-DTA/DSC thermogravimetric analyzer (SETARAM, Caluire, France). The measurements were carried out in corundum crucibles in the temperature range of 30–1000 °C in an argon atmosphere. The flow rates of the protective and purge gases were 20 and 50 mL/min, respectively. The experiments were performed at heating rates of 5.0, 7.5, 10.0, and 12.5 °C/min. The weight of the samples was 10.0 ± 0.5 mg. Each measurement was repeated three times (*n* = 3) to ensure reproducibility and statistical processing.

The results were processed, and graphs were constructed in OriginPro 9.0 and Python 3.10 (Anaconda) using the NumPy, SciPy, and Matplotlib (3.10.1) libraries. Friedman, Ozawa-Flynn-Wall, and Non-Parametric Kinetic (NPK) model-free methods were used to calculate kinetic parameters. Model analysis was performed using the Shestak-Berggren equation, which allowed us to evaluate the characteristics of thermodestruction mechanisms.

The statistical significance of the differences between the approaches was tested using Student’s *t*-test and one-way analysis of variance (ANOVA) at a significance level of *p* < 0.05, calculated for each degree of conversion α.

#### 2.3.1. Method for Evaluating Thermal Stability Under Cyclic Temperature Exposure

To evaluate the resistance of hydrogels to repeated thermal exposure, a cyclic thermogravimetric analysis (TGA) procedure was developed and implemented.

Each temperature cycle consisted of four sequential stages:

- heating from 30 to 100 °C at a rate of 10 °C min^−1^;
- an isothermal hold at 100 °C for 10 min;
- cooling down to 30 °C at a rate of 5 °C min^−1^;
- stabilization at 30 °C for 10 min to achieve thermal equilibrium.

This temperature profile was repeated for 20 consecutive cycles, while the sample mass was continuously recorded using thermogravimetric analysis (TG). The obtained data made it possible to evaluate the thermal stability, mass-loss dynamics, and reversibility of sorption–desorption processes under conditions of cyclic thermal loading.

#### 2.3.2. Evaluation of Hydrogel Dehydration Kinetics Using the Fick Model

Isothermal dehydration was carried out at a temperature of 80 °C for one hour. Samples weighing 10–15 mg were placed in aluminum crucibles and heated in an argon atmosphere at a flow rate of 20 mL/min. The mass was recorded at 1 min intervals with an accuracy of ±0.1 μg. For dehydration analysis, we used the Fick model [20], which describes the molecular diffusion of moisture in a confined plate. The mass values *M_t_* were normalized to the mass of the absolutely dry sample *M_∞_*. After that the following dependencies were constructed:

(1) $$\frac{M_{t}}{M_{\infty }},\;ln\left(1-\frac{M_{t}}{M_{\infty }}\right)$$

The initial stage (up to ~30% mass loss) was approximated by a linear equation:

(2) $$ln\left(1-\frac{M_{t}}{M_{\infty }}\right)=-kt+C$$

where *M_t_* is the mass of the sample at time *t*; *M_∞_* is the mass of the dry sample; *k* is the effective dehydration rate (min^−1^); *C* is the integration constant.

The values of the diffusion coefficient *D* were calculated using the formula:

(3) $$D=\frac{k\cdot L^{2}}{\pi^{2}}$$

where L—half the thickness of the sample.

In this work, samples with a thickness of 3*L* = 3 mm were used, i.e., *L* = 1.0 × 10^−3^ m.

The quality of the approximation was assessed using the coefficient of determination *R^2^*, which exceeded 0.94, confirming the adequacy of the Fick model at the initial stage of dehydration. All calculations and graphical visualization were performed using OriginPro 9.0 and Python (NumPy, SciPy, Matplotlib). Mean values and standard deviations for three independent measurements (*n* = 3) were used to evaluate the accuracy of the parameters.

### 2.4. Cryogenic Scanning Electron Microscopy (cryo-SEM)

The morphology of hydrogels was studied using cryogenic low-vacuum scanning electron microscopy (cryo-LVSEM or cryo-SEM). The experiments were performed using a MAIA3 (TESCAN, Brno, Czech Republic) field emission scanning electron microscope equipped with a secondary electron (SE) detector and a Peltier cooling stage. Details of the experiments are described elsewhere [21]. Briefly, a small piece of hydrogel sample (approximately 2 mm × 2 mm × 2 mm) was rapidly frozen in liquid nitrogen and transferred to a Peltier stage (cooled to −15 °C) in the vacuum chamber of the microscope. The frozen sample was fixed on the Peltier stage with a drop of water (the drop of water freezes within a few seconds, fixing the sample on the stage), and the thin top layer of the sample was cut off with a razor blade cooled with liquid nitrogen (this removes the top layer of the frozen sample, which can deform and/or become covered with ice microcrystals). The morphology of the hydrogel was observed in low vacuum mode (chamber pressure 50 Pa) using a low vacuum SE detector at an accelerating voltage of 15 kV.

## 3. Results and Discussion

**Figure 3:**
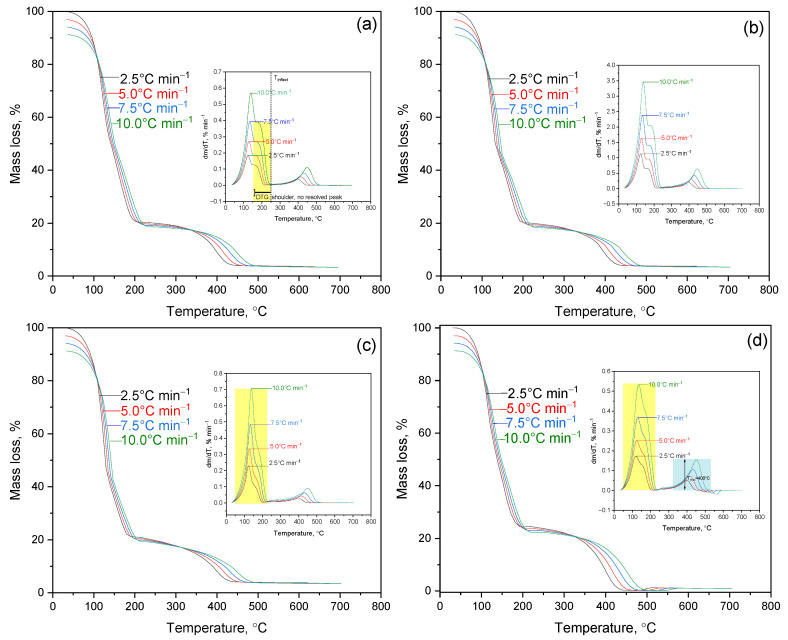
TGA-curves of IPN hydrogel of PVA/PEGDA–PEGMA (PEGMA 11 wt.% in all samples, H_2_O 75–81 wt.%), compositions: (**a**) 1/7; (**b**) 2/7; (**c**) 3/7; (**d**) 3/11 (PVA/PEGDA wt.%).

Figure 3 presents the TGA/DTG curves of PVA/PEGDA–PEGMA hydrogels (PEGMA 11 wt.% in all samples, H_2_O 75–81 wt.%) with the following compositions: (a) 1/7, (b) 2/7, (c) 3/7, and (d) 3/11 (PVA/PEGDA wt.%).

Sample (a), containing 1 wt.% PVA and 7 wt.% PEGDA, exhibits the lowest crosslink density among the studied systems, resulting in the loosest polymer network. The first temperature interval (50–160 °C) corresponds to dehydration with a mass loss of 77.8%, while T_max_ = 140 °C (DTG) reflects the removal of free and capillary water, i.e., moisture not bound to the polymer matrix. The second interval (160–240 °C) is weakly pronounced (Δm = 3.6%) and appears on the DTG curve only as a slight baseline inflection, corresponding to the onset of intramolecular dehydration of PVA and thermolysis of unstable side groups. These initial reactions indicate the beginning of chemical degradation, although still at a relatively low intensity. In samples (a) and (b), these processes are detected as a distinct but minor effect, whereas in (c) and (d) they overlap with the main decomposition stage. The principal stage of thermodestruction occurs between 376 and 492 °C with T_max_ = 445 °C, and the DTG peak half-width (FWHM) of 68.1 °C indicates a relatively prolonged degradation process. Such a broad profile reflects gradual decomposition, which is favorable for systems designed for controlled release or slow degradation. The residual mass above 450 °C is only 0.4%, confirming the absence of thermally stable carbonaceous residue—an important characteristic for assessing long-term stability and “clean” thermal decomposition.

A comparison with sample (b), containing 2 wt.% PVA at the same PEGDA and PEGMA contents, shows that the additional introduction of hydrophilic fragments enhances moisture retention. The dehydration T_max_ shifts to a higher temperature (187.7 °C), and the dehydration signal broadens (50–250 °C), with Δm = 75.0%. This behavior indicates stronger water binding, which is valuable for hydrogel matrices requiring moisture-retaining functionality. The main decomposition occurs between 376 and 488 °C with T_max_ = 443 °C. The process becomes more localized, as evidenced by a narrower FWHM of 59.6 °C, suggesting a faster and more concentrated degradation. This may be advantageous for rapid-release systems but less favorable for long-term applications. The residual mass is 0.5%.

Sample (c), with 3 wt.% PVA and 7 wt.% PEGDA, demonstrates more intense dehydration (Δm = 83.9%) with T_max_ = 135 °C, indicating stronger moisture binding and higher hydrophilicity. The main decomposition occurs in the range of 383–487 °C with T_max_ = 453 °C, while the DTG peak exhibits a significant half-width (FWHM = 80.3 °C), suggesting a more extended and gradual degradation process. Such a broad DTG profile indicates enhanced resistance to thermal fluctuations, which is beneficial for sterilization and storage stability. The residual mass is 0.6%.

Sample (d), containing 3 wt.% PVA and 11 wt.% PEGDA (PEGMA = 11 wt.% and water = 75 wt.% kept constant), exhibits markedly different behavior. The main decomposition stage shifts to a lower temperature range (240–510 °C), with the maximum decomposition rate observed at T_max_ = 400 °C. The DTG peak is both broad and intense (FWHM = 87.3 °C), and the residual mass is minimal (0.3%). This behavior reflects local heat accumulation and accelerated degradation at high crosslink densities. While such dense networks enhance mechanical robustness prior to breakdown, they can also degrade more rapidly once decomposition begins. According to the literature [9,22], high crosslink density restricts heat transfer and limits the diffusion of volatile degradation products, leading to localized overheating and accelerated degradation—an effect that likely occurs in this system as well. This finding is of practical relevance, as it indicates that overly rigid hydrogel networks may undergo faster breakdown once decomposition is initiated.

The key parameters characterizing the thermal behavior of samples (a–d) are summarized in Table 1.

The observed differences in the thermal behavior of PVA/PEGDA–PEGMA hydrogels (at a constant PEGMA content of 11 wt.% and water content of 75–81 wt.%) for the compositions (a) 1/7, (b) 2/7, (c) 3/7, and (d) 3/11 (wt.% PVA/PEGDA) are determined by the specific degradation pathways of the polymer matrix components. Poly(vinyl alcohol) (PVA) undergoes thermodegradation initiated by hydroxyl groups, which promotes depolymerization accompanied by the formation of acetic acid and carbonyl-containing products [23,24]. In other words, the presence of hydroxyl groups renders PVA thermally sensitive. Poly(ethylene glycol) diacrylate (PEGDA), in contrast, decomposes primarily through the hydrolysis of ester linkages and subsequent β-scission upon heating, yielding oligomeric fragments and volatile products [25]. Thus, PEGDA behaves as a typical polyester, with hydrolysis preceding chain cleavage during degradation. An increase in the PEGDA fraction enhances the crosslink density and decreases the water-binding capacity of the hydrogel (the network becomes stiffer and retains less moisture), whereas higher PVA content promotes water uptake and facilitates hydrolytic processes (the network becomes more open and “absorptive”). These structural and compositional factors explain the observed shifts in decomposition temperatures and the characteristic shapes of the DTG peaks (Figure 3). Broad DTG peaks correspond to gradual and distributed degradation, while narrow peaks indicate rapid and localized decomposition events. Similar degradation patterns have been reported for other interpenetrating polymer networks based on PVA and polyacrylates, where hydrophilicity and crosslink density are key factors determining thermal stability. Hence, the obtained data are consistent with previously described degradation mechanisms of analogous polymeric systems.

It should be emphasized that the parameters T_max_ and DTG peak half-width (FWHM) have practical significance for predicting the operational performance of the studied materials. A shift in T_max_ toward higher temperatures reflects improved thermal stability of the polymer network, which is crucial for technological processes such as hydrogel drying or sterilization and for long-term storage. The FWHM value, in turn, characterizes the nature of the degradation process: a broader peak indicates gradual and uniform decomposition, whereas a narrower peak corresponds to abrupt, localized breakdown—potentially associated with localized heat accumulation. From a practical perspective, these differences provide insight into the stability and degradation kinetics of the materials, which is particularly relevant for their use as sorbents or carriers of biologically active compounds, where controlled decomposition or “clean” burnout without carbon residue may be desirable.

In addition to thermogravimetric results, supplementary rheological, structural, and morphological data are provided in Supplementary Materials S3 (Figures S3–S7). These studies confirm the sufficient mechanical strength, stable microstructure, and ordered phase morphology of the IPN hydrogels—demonstrating that the materials are not only thermally stable but also structurally robust under mechanical loading, thereby highlighting their potential for biomedical applications.

For samples (b) and (c), the variations in T_max_ and water-binding capacity are consistent with the results summarized in Table 1, confirming the critical role of PVA content and crosslink density in determining water retention. In contrast, an increase in PEGDA concentration to 11 wt.% at a fixed 3 wt.% PVA (sample d) leads to the formation of a denser network, which limits the amount of bound water and shifts the main decomposition stage to a lower temperature range (300–400 °C). This behavior suggests that overly rigid networks may degrade earlier due to the accumulation of internal stresses and restricted diffusion processes.

Thermal degradation of polymer materials is directly linked to their operational reliability and technological performance [22]. For hydrogels [26]—used as sorbents, drug carriers, and functional biomaterials—water retention capacity and resistance to sterilization treatments are particularly important, as these properties determine whether the material can withstand drying, sterilization, or long-term storage. These characteristics are governed by thermal stability, which defines the synthesis parameters, storage conditions, and practical usability of the material [17].

Activation energy (*E_a_*) is one of the key parameters characterizing the thermal stability of polymers. Analyzing its variation as a function of the degree of conversion (α) makes it possible to identify the sequence of decomposition stages—i.e., which bonds break first and which remain the most stable—to determine the most vulnerable structural elements, and to evaluate the influence of polymer architecture and crosslink density on the durability of the matrix.

Isoconversional methods, including the Friedman [27] and Ozawa–Flynn–Wall (OFW) [28,29] approaches, enable the calculation of *E_a_* without prior assumptions about the reaction mechanism. In other words, they allow one to analyze the kinetics without presuming a specific reaction pathway in advance. This makes such methods particularly effective for studying complex, multicomponent systems such as hydrogels.

It should be emphasized that similar studies on PVA/PEGDA–PEGMA hydrogels (PEGMA 11 wt.% in all samples, H_2_O 75–81 wt.%) have not been conducted previously. For the first time, samples with different degrees of crosslinking were compared by calculating the distribution of activation energy as a function of α, thus revealing how the energetic barrier changes step-by-step during the degradation process. This approach made it possible to identify the critical stages of thermal degradation, determine the influence of PVA content and PEGDA concentration on thermal stability, and clarify the degradation mechanism. The combined application of the Friedman and OFW methods provided a comprehensive description of thermolysis kinetics, significantly enhancing the understanding of hydrogel stability in this system and offering new possibilities for targeted optimization of their properties.

**Figure 4:**
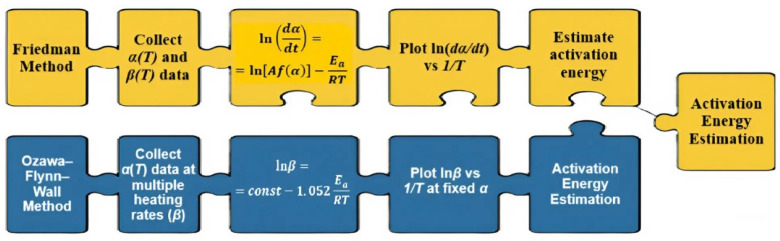
Graphical illustration of the differences between the differential (Friedman) and integral (OFW) methods.

The kinetic analysis was performed using the Friedman and Ozawa–Flynn–Wall (OFW) isoconversional methods. Figure 4 schematically illustrates the difference between these approaches: the Friedman method is based on the differential form of the Arrhenius equation and analyzes the dependence of ln(dα/dt) on 1/T at fixed conversion degrees (i.e., focusing on the reaction rate at specific stages), whereas the OFW method applies an integral form and examines the dependence of lnβ on 1/T at different heating rates (i.e., considering the overall reaction process). The combined use of these complementary methods ensures the consistency of results and provides a more complete and reliable description of the thermal degradation mechanism of hydrogels.

**Figure 5:**
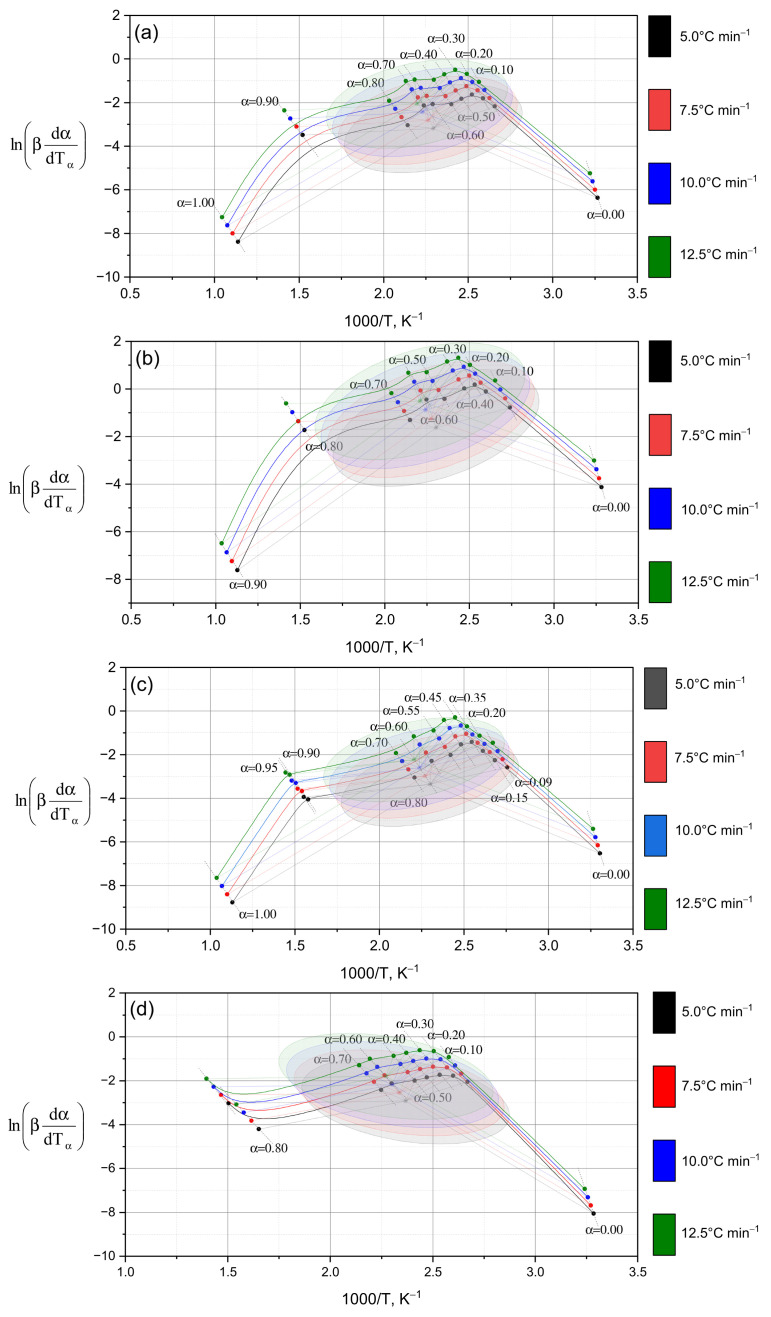
Isoconversion analysis curves (Friedman method) for IPN hydrogels of PVA/PEGDA–PEGMA (PEGMA 11 wt.% in all samples, H_2_O 75–81 wt.%): (**a**) 1/7; (**b**) 2/7; (**c**) 3/7; (**d**) 3/11 (PVA/PEGDA wt.%).

Following the schematic diagram (Figure 4), which illustrates the distinctions between the differential (Friedman) and integral (Ozawa–Flynn–Wall, OFW) approaches, the results of their application to the experimental data are presented below. Figure 5 shows the kinetic dependencies constructed for different heating rates (from 5 °C to 12.5 °C), which made it possible to calculate the distribution of activation energy across the conversion degrees.

The dependencies of ln(βdα/dt) on 1/T for various conversion degrees (α = 0.0–1.0) are shown in Figure 5. As α increases, the curves shift and reach their maximum values within the central ellipsoidal region (α = 0.1–0.8), followed by a subsequent decrease. This behavior reflects a general kinetic trend: the activation energy decreases with increasing conversion degree [30]—in other words, as the reaction progresses, less energy is required to sustain it.

This trend is quantitatively confirmed by the data in Table 2, where a consistent decrease in activation energy from α = 0.1 to α = 0.9 is observed for all samples studied (a–e). For sample (a) (Figure 5a), the Friedman method yields values decreasing from 101.87 to 86.86 kJ·mol^−1^ (101.87 → 95.09 → 92.66 → 87.73 → 86.86), fully consistent with the results obtained using the OFW method. In the initial stages (α = 0.1–0.3), the maximum activation energy values (~100–107 kJ·mol^−1^) are recorded in all cases, reflecting the destruction of the most stable chemical bonds within the polymer structure [31] (these correspond to the “strongest building blocks” of the material). As the process proceeds (α = 0.5–0.8), the activation energy decreases to ~84–87 kJ·mol^−1^, indicating the involvement of less stable bonds (that is, “weaker links” begin to decompose).

It should be noted that the rate of activation energy decrease varies between samples. In sample (b) (Figure 5b), a sharp decline occurs between α = 0.1 (107.47 kJ·mol^−1^) and α = 0.3 (95.54 kJ·mol^−1^), while in sample (d) (Figure 5d), the decrease is more gradual. Such heterogeneity confirms the multistage nature of the decomposition process: each stage proceeds via its own mechanism. A more detailed comparison of samples (c) and (d) (Figure 5c,d) shows that at the initial stage, both exhibit nearly identical activation energy values (~103 kJ·mol^−1^ by Friedman and ~100 kJ·mol^−1^ by OFW). However, at intermediate stages (α = 0.3–0.5), sample (d) shows higher values (95.78 kJ·mol^−1^ by OFW vs. 90.44 kJ·mol^−1^ for sample (c)), suggesting the participation of stronger bonds (its network “resists” decomposition longer). At later stages (α = 0.7–0.9), activation energy decreases more sharply in sample (c), while in sample (d) the values remain comparatively high—indicating that this material still requires greater energy input for degradation. Thus, the differences in activation energy evolution point to distinct decomposition mechanisms: sample (c) transitions to less energy-intensive stages, whereas sample (d) maintains a high energy barrier.

To verify the consistency between the Friedman and Ozawa–Flynn–Wall methods, statistical analysis was performed (Table 3). According to the ANOVA results, no significant differences between the two methods were detected (*p* > 0.05 for all samples), which can be attributed to the relatively high data dispersion and averaging over the entire sample set (in other words, the “spread” of data masks local variations). However, a paired *t*-test, which is more sensitive to local differences [32], revealed statistically significant discrepancies (*p* < 0.05) for samples (a–c), whereas for sample (d) no significant difference was found (*p* = 0.20). Hence, the distinction between the two statistical outcomes reflects the differing sensitivities of the methods: ANOVA captures general trends (“a coarse filter”), while the *t*-test detects subtle sample-level variations (“fine adjustment”). The convergence of results for sample (d) indicates a stable and uniform decomposition mechanism, whereas the discrepancies observed for samples (a–c) confirm the kinetic complexity and multistage character of their thermal degradation.

Thus, the data presented in Figure 5 and Table 2 and Table 3 demonstrate that the reactions under study do not follow a single kinetic model. The variation in activation energy as a function of the conversion degree (α) indicates a change in the reaction mechanism as the process progresses (i.e., the decomposition pathway evolves over time), which justifies the application of model-independent isoconversional methods (Friedman and Ozawa–Flynn–Wall) for an accurate description of the reaction kinetics.

The differences observed in the thermal parameters of the TGA curves (Figure 3) are reflected in the distribution of activation energy (*E_a_*) calculated using isoconversional methods. As shown in the subsequent analysis, an increase in PEGDA content leads to a higher activation energy barrier for thermal degradation, which correlates with the enhanced crosslink density and the restricted diffusion of moisture within the polymer matrix. In contrast, samples with higher PVA content exhibit lower activation energy values, attributed to their higher hydrophilicity and the presence of hydrolyzable fragments that facilitate the initiation of decomposition.

However, the use of isoconversional methods alone does not always provide an accurate description of the behavior of multicomponent or modified systems, where several decomposition stages often overlap, meaning that multiple reactions occur simultaneously and influence each other. In such cases, it becomes particularly important to apply approaches that do not require an a priori selection of a kinetic model and are capable of distinguishing overlapping processes.

To study the thermal degradation of complex systems, model-free kinetic methods are considered highly effective, as they do not rely on predefined reaction mechanisms—allowing the data to “speak for themselves” without being constrained by prior model assumptions. One of the most powerful tools for such analysis is the nonparametric kinetics (NPK) method [33,34], which is based on mathematical processing of thermogravimetric data obtained at different heating rates, i.e., by comparing mass-loss curves recorded under varying thermal conditions. Unlike traditional isoconversional approaches (e.g., Friedman or Ozawa–Flynn–Wall), the NPK method enables the effective separation of overlapping stages and thus provides a more detailed interpretation of multicomponent processes. In other words, it can “deconvolute” multiple parallel reactions and reveal their individual contributions.

The NPK algorithm represents experimental data as a matrix containing the degree of conversion and the corresponding reaction rates—essentially a data array that reflects how fast the reaction proceeds at different temperatures. Applying singular value decomposition (SVD) to this matrix allows it to be decomposed into principal components, each corresponding to an individual kinetic process, analogous to identifying dominant patterns in complex datasets. This approach facilitates the separation of overlapping decomposition stages, which is particularly valuable when studying modified systems, and ensures more accurate data approximation with reduced errors compared to model-based methods, as it eliminates distortions caused by incorrect model assumptions.

**Figure 6:**
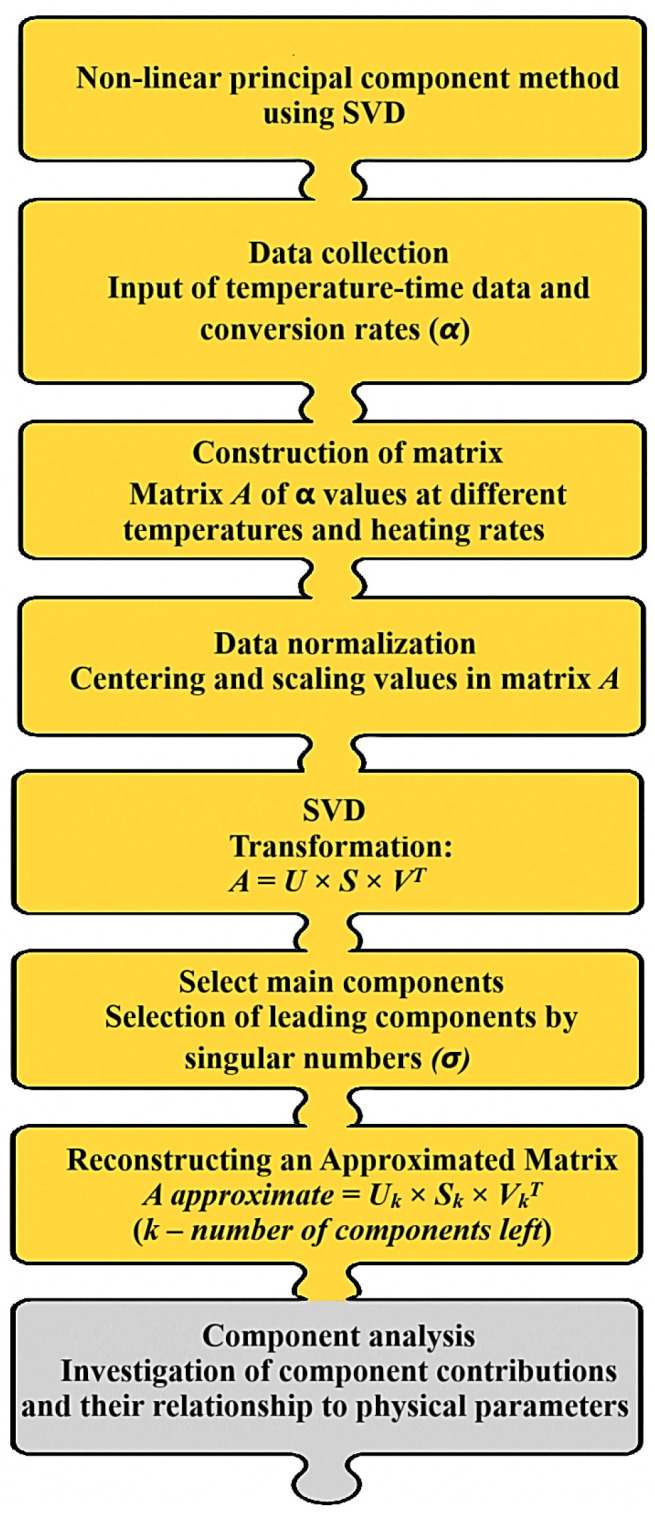
Block diagram of the application of SVD within the framework of the nonparametric kinetics (NPK) method for the analysis of thermogravimetric data.

For clarity, Figure 6 presents a schematic diagram of the PCA-based analysis, illustrating the formation of the initial data matrix, SVD, selection of principal components, and evaluation of their contribution to the overall reaction process—that is, how the method extracts individual reaction mechanisms from the experimental data.

**Figure 7:**
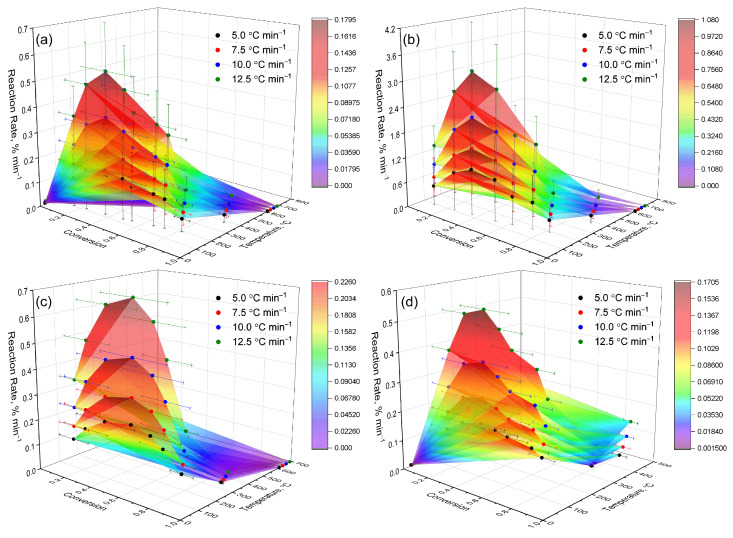
Three-dimensional dependencies of the reaction rate (dα/dT) on temperature (T) and degree of conversion (α) at heating rates of 5.0, 7.5, 10.0, and 12.5 °C·min^−1^. The kinetic profiles of the thermal decomposition of IPN hydrogels PVA/PEGDA–PEGMA (PEGMA 11 wt.% in all samples, H_2_O 75–81 wt.%) are shown: (**a**) 1/7; (**b**) 2/7; (**c**) 3/7; (**d**) 3/11 (PVA/PEGDA wt.%).

For a more detailed analysis of the thermokinetic characteristics of the studied systems, the dependence of the decomposition rate (dα/dT) on temperature (T) and the degree of conversion (α) was visualized in the form of three-dimensional plots (Figure 7), illustrating how rapidly the material decomposes at different temperatures and stages of the process. The resulting surfaces represent visual “kinetic landscapes” that allow us to track the dynamics of thermal decomposition across the entire range of temperatures and conversions—in other words, they reveal where the reaction is most active and how it evolves during heating. This approach makes it possible to identify the characteristic features of each sample’s behavior, including shifts in the temperature ranges of active decomposition and the differences associated with structural modifications and complexation effects [35], such as the influence of polymer composition or intermolecular interactions on network stability.

The subsequent analysis provides a step-by-step description of the kinetic characteristics identified for each of the samples. Figure 7a–d show three-dimensional dependencies of the reaction rate (dα/dT) on temperature (T) and conversion degree (α) at heating rates of 5.0, 7.5, 10.0, and 12.5 °C·min^−1^. The construction of 3D surfaces enabled the identification of distinct features of the thermal decomposition of hydrogels, clearly illustrating the main zones of activity and their shifts depending on the heating rate. All numerical values of the maximum decomposition rates were obtained from the experimental 3D dependencies (Figure 7).

The three-dimensional plots revealed fundamental differences among the samples. Sample (a) exhibited moderate activity and a simplified kinetic pattern, with decomposition occurring predominantly in a single stage without significant process overlap. Sample (b) demonstrated the highest reactivity and a multistage mechanism, attributed to the increased mobility of the polymer network—its less dense structure facilitates chain movement and accelerates the reaction. Sample (c) showed a narrow zone of activity and rapid attenuation of the process, indicating diffusion limitations and high thermal stability, as heat and volatile products are released more slowly while the structure remains stable. Sample (d) occupied an intermediate position: the reaction proceeded more gradually and over a wider range of temperatures, reflecting a less compact structure and the participation of secondary processes, such as side reactions or the gradual decomposition of residual fragments.

**Figure 8:**
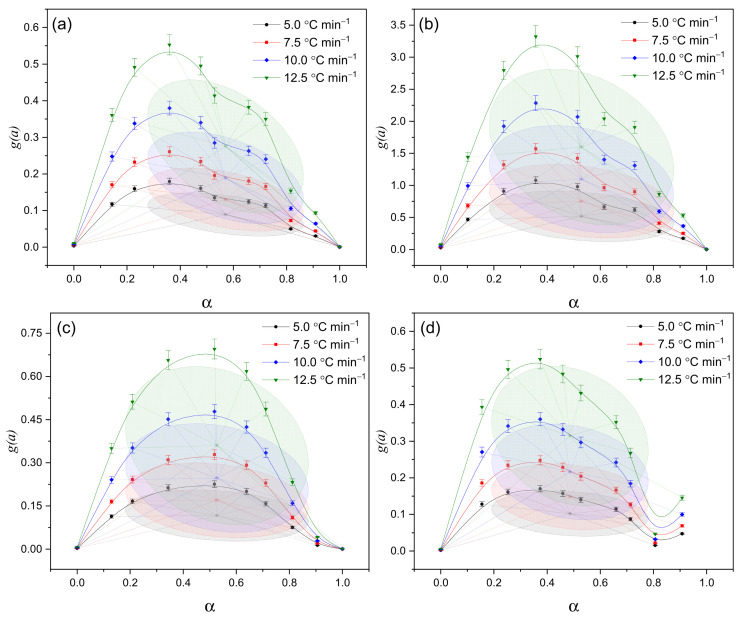
Approximation of experimental data in g(α)–α coordinates at heating rates of 5.0; 7.5; 10.0, and 12.5 °C·min^−1^, illustrating the features of the decomposition mechanism of IPN hydrogels PVA/PEGDA–PEGMA (PEGMA 11 wt.% in all samples, H_2_O 75–81 wt.%): (**a**) 1/7; (**b**) 2/7; (**c**) 3/7; (**d**) 3/11 (PVA/PEGDA wt.%).

The g(α)–α approximation data (Figure 8) confirm the previously identified distinctions between the samples. In sample (c), the process is diffusion-limited, meaning that the movement of volatile products slows down the overall reaction rate. In sample (d), the reaction proceeds more gradually and remains active over an extended range of conversion, indicating a prolonged and continuous decomposition process. In contrast, sample (a) exhibits a simplified kinetic behavior with moderate reactivity, corresponding to an almost single-stage process with minimal complexity.

**Figure 9:**
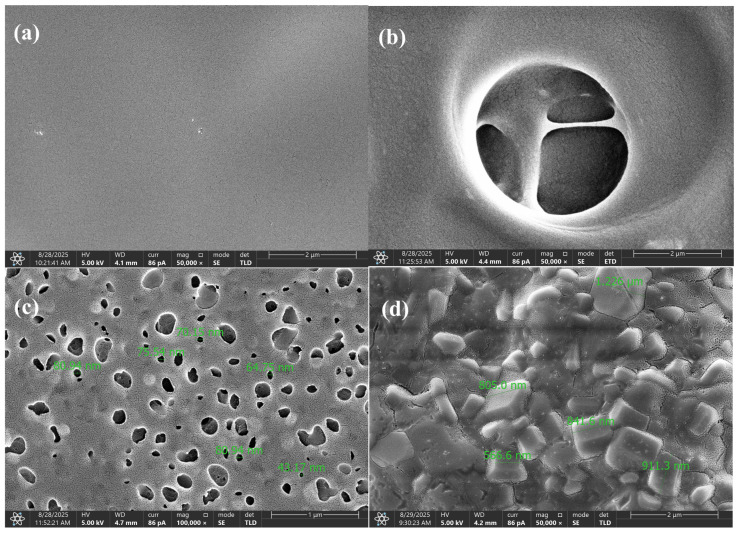
SEM images of the PVA/PEGDA–PEGMA composition (PEGMA 11 wt.% in all samples, H_2_O 75–81 wt.%, PVA/PEGDA = 2:7) at ×50,000 magnification after heat treatment at different temperatures (**a**) 150, (**b**) 250, (**c**) 450, (**d**) 600 °C.

Morphological transformations (Figure 9) have a direct impact on the reaction kinetics and activation energy. At the early stage (150 °C), the dense structure of the composition inhibits the diffusion of volatile products, thereby requiring higher activation energy to initiate destructive reactions—i.e., the material must absorb more heat before decomposition begins. In the range of 250–450 °C, the formation of gas bubbles and the development of nanoporous domains significantly enhance heat transfer and gas diffusion, lowering the energy barrier and accelerating the decomposition of the polymer matrix. As the sample becomes more porous, it also becomes more reactive.

At approximately 600 °C, a carbonized, crystallite-like structure with high thermal stability is formed; its further transformation again demands increased activation energy, indicating that the residual material becomes more resistant to degradation. Thus, the evolution of morphology exhibits a nonlinear behavior: from increased energy consumption during the initial dehydration phase (associated with a compact structure), to a decrease in the active zone of gas release and pore formation (corresponding to structural loosening), followed by another increase during the formation of a rigid carbon matrix.

The Supplementary Materials S4 and S5 (Figures S8–S12) include IR spectra and SEM micrographs of the composition at different temperatures, which confirm these structural transitions by clearly demonstrating the emergence of porosity and the gradual carbonization of the material.

These observations are consistent with the results of the parametric analysis (Table 4), particularly regarding the activation energy and pre-exponential factor, which describe how easily the reaction is initiated. For sample (a), the activation energy was determined to be 102.70 ± 0.38 kJ·mol^−1^ with an average pre-exponential factor of $\overset{-}{A}$ = (29.30 ± 0.20) × 10^13^ s^−1^, indicating a pronounced kinetic barrier—i.e., the reaction requires a significant amount of energy to initiate, and the material remains relatively stable at moderate temperatures.

For sample (b), which exhibited the highest decomposition rate, the lower average activation energy (93.67 ± 0.45 kJ·mol^−1^) combined with a smaller pre-exponential factor ($\overset{-}{A}$ = (1.45 ± 0.05) × 10^13^ s^−1^) suggests a more energetically accessible but less stable process, meaning that the material decomposes more readily upon heating.

In contrast, sample (c), characterized by a low decomposition rate and sharp decay, shows the minimum pre-exponential factor ($\overset{-}{A}$ = (0.05 ± 0.01) × 10^13^ s^−1^), which corresponds to limited kinetic activity and a narrow temperature range of decomposition—i.e., the reaction proceeds rapidly within a short interval and then ceases.

Sample (d), on the other hand, demonstrates an activation energy of 103.59 ± 0.26 kJ·mol^−1^ and maintains reactivity over a broader range of conversion degrees, consistent with its smooth profile on the 3D kinetic maps. This indicates a gradual, uniform degradation process and structural stability under heating conditions.

Additionally, the parameters of the Šesták–Berggren model support the identified distinctions: for sample (c), the values m = 0.48 and n = 0.45 indicate strong diffusion limitations and a localized nature of the reaction (i.e., the process occurs in confined regions and is restricted by molecular mobility). In contrast, for sample (d) (m = 0.62; n = 1.05), the kinetics correspond to a process taking place over a broader range of conversion degrees and temperatures, suggesting a smoother and more prolonged decomposition.

In the present study, a comprehensive approach was applied to the kinetic analysis of the thermal decomposition of IPN hydrogels, combining both model-free and model-based methods (i.e., approaches that either assume or do not assume a predefined reaction mechanism). Each selected method served a specific analytical purpose aimed at constructing an integrated understanding of the degradation mechanisms:

The Friedman and Ozawa–Flynn–Wall (OFW) models were used to evaluate the variation in activation energy (*E_a_*) as a function of the conversion degree (α), allowing us to trace how the energetic barrier changes throughout the decomposition process and to identify its multistage and kinetically complex character.

The NPK method was applied to determine the functional dependence f(α) without the need to predefine the mechanism—a crucial advantage in systems with overlapping reactions where individual processes cannot be easily distinguished.

The Šesták–Berggren model was employed to describe autocatalytic effects (where the reaction products promote further decomposition), which are characteristic of highly crosslinked structures and are not revealed by other kinetic models.

For clarity, Supplementary Materials S6 provides a summary table (Table S1) outlining the purpose, type, and analytical significance of each applied model within the context of this study.

Thus, the combined use of these approaches provided a multilevel interpretation of the thermal degradation mechanisms—from the identification of individual stages and activation parameters to the formal dependencies of the reaction rate.

Statistical analysis (Table 4) confirmed these conclusions. The analysis of variance (ANOVA) revealed no significant differences between the Friedman and Šesták–Berggren methods (*p* > 0.05 for all samples). However, the paired *t*-test indicated that for samples (a–c), the differences are statistically significant (*p* < 0.05), whereas for sample (d) no significant difference was observed (*p* = 0.20). The convergence of results for sample (d) demonstrates a high degree of agreement between the two approaches and confirms a stable, uniform decomposition mechanism (i.e., the reaction proceeds evenly without abrupt transitions). In contrast, the discrepancies observed for samples (a–c) reflect the complexity and multistage nature of their kinetics, where different reactions become activated within distinct temperature ranges. These findings are consistent with the data obtained from the three-dimensional plots (Figure 8) and the *g*(α)–α approximations (Figure 6).

Thus, qualitative analysis based on 3D dependencies and quantitative evaluations using the NPK and Šesták–Berggren methods provide a consistent picture of the kinetic behavior of the studied hydrogels. Sample (a) follows a relatively simple kinetic model with moderate activity, indicating that decomposition occurs in one or two stages without significant overlap of processes. Sample (b) exhibits high reactivity but limited thermal stability—it decomposes rapidly but is less resistant to further heating. In contrast, samples (c) and (d) demonstrate higher stability, differing in their kinetic mechanisms and structural factors: sample (c) shows a narrow kinetic window and diffusion-controlled decomposition (slow degradation limited by molecular mobility), while sample (d) displays a more extended process with a broader temperature range of activity and prolonged degradation.

The practical significance of these differences lies in the ability to tailor hydrogel composition to specific functional requirements. For instance, sample (b), characterized by high reactivity, is suitable for applications that demand rapid or programmable degradation, such as drug delivery systems, temporary implants, or materials with tunable degradation times. Sample (a), which exhibits moderate activity and relatively high activation energy, is advantageous for applications requiring a balance between degradation rate and thermal stability, such as protective coatings or thermoresistant polymer matrices. The enhanced stability of samples (c) and (d) makes them promising candidates for long-term use in heat-resistant hydrogels, barrier coatings, and biomedical implants, where high mechanical and thermal endurance are essential.

**Figure 10:**
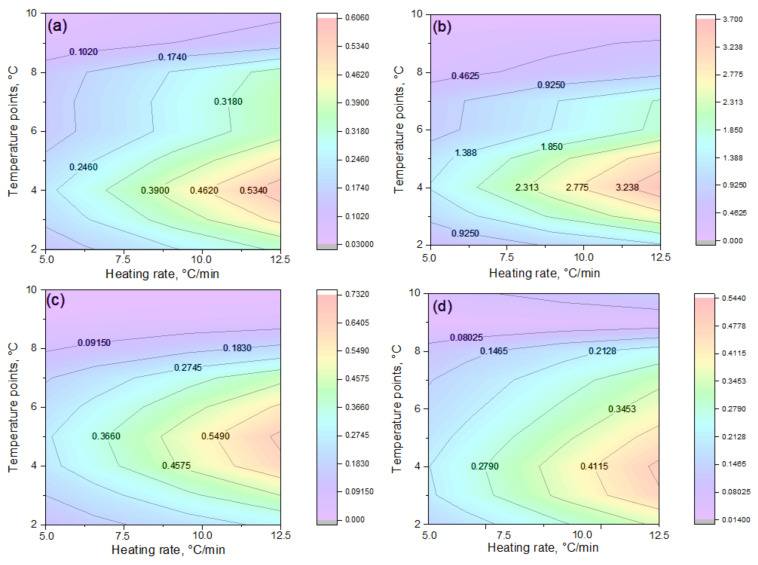
Thermal maps of dα/dt distribution in T–β coordinates at 5.0; 7.5; 10.0, and 12.5 °C·min^−1^ for IPN hydrogels of PVA/PEGDA–PEGMA (PEGMA 11 wt.% in all samples, H_2_O 75–81 wt.%): (**a**) 1/7; (**b**) 2/7; (**c**) 3/7; (**d**) 3/11 (PVA/PEGDA wt.%). The numerical values on the maps (e.g., 0.1020; 0.2460; 0.5340) reflect the decomposition rate (dα/dt, %·min^−1^) and correspond to isolines of equal reaction activity. The color scale on the right visualizes the intensity of the process, allowing temperature ranges to be correlated with the level of kinetic activity.

Figure 10 presents thermal maps of the reaction rate distribution (dα/dt) plotted in “temperature–heating rate” coordinates for hydrogels of different compositions. For sample (a), the zone of maximum activity is concentrated within a narrow temperature range at high heating rates, while at lower rates, the process proceeds more gradually and is more prolonged, indicating kinetics of moderate intensity with a limited number of stages. The heat maps confirm the previously observed trends: sample (b) exhibits maximum reactivity and a multistage decomposition behavior, suggesting the superposition of several concurrent reactions. In sample (c), the activity zone is confined and decays rapidly, consistent with its dense network structure and reduced chain mobility that hinder degradation. Sample (d) demonstrates a broader range of thermal activity with a gradual decline in reaction rate, indicative of a prolonged decomposition mechanism. Sample (a) occupies an intermediate position, combining moderate activity with a simple kinetic profile, and can thus be regarded as a transitional case between fast- and slow-degrading systems.

A comparative analysis of thermal maps (Figure 10) confirms the conclusions obtained from the results of 3D dependencies, *g(α)–α* approximation, and parametric analysis (Table 3 and Table 4). The heat maps (Figure 10) confirmed that sample (b) has the maximum reactivity and multistage decomposition (see description above), while sample (c) demonstrates a narrow activity window and rapid decay (see Figure 7 and Figure 8), which is consistent with its dense lattice. Sample (d) is characterized by a more prolonged destruction process and preservation of activity in the later stages, which is consistent with its high activation energy and Šesták–Berggren model parameters. Sample (a) occupies an intermediate position, combining a moderate decomposition rate with a relatively simple kinetic picture.

Thus, the thermal map data not only illustrate the temperature-dependent reaction activity of the hydrogels but also provide additional confirmation of the structurally determined differences in their kinetic behavior, namely the relationship between network density, chain mobility, and degradation rate.

Thermokinetic analysis (Figure 7, Figure 8, Figure 9 and Figure 10, Table 3 and Table 4) revealed significant differences in the stability and decomposition mechanism of hydrogels. To compare these results with the behavior in a humid environment, the dehydration kinetics characterizing the moisture retention capacity and structure of the polymer network were additionally studied. Quantitative analysis was performed using linear approximation of experimental data in time coordinates based on the analytical solution of Fick’s equation. The slope of the approximated straight-line k was used to calculate the effective diffusion coefficient D using the expression:

(4) $$D=\frac{k\cdot L^{2}}{\pi^{2}}$$

where $L=\frac{1}{2}$ sample thickness in meters. All calculated parameters—slope *k*, coefficient of determination *R*^2^, and corresponding *D* values—are summarized in Table 5.

**Figure 11:**
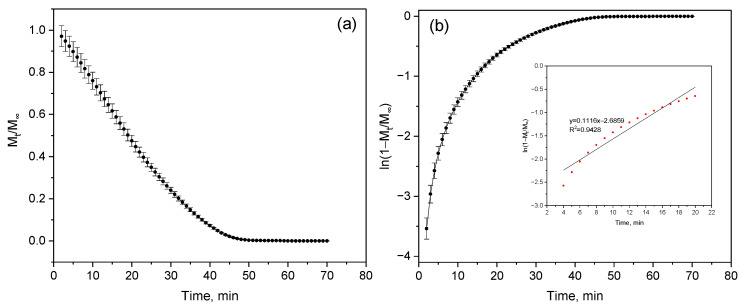

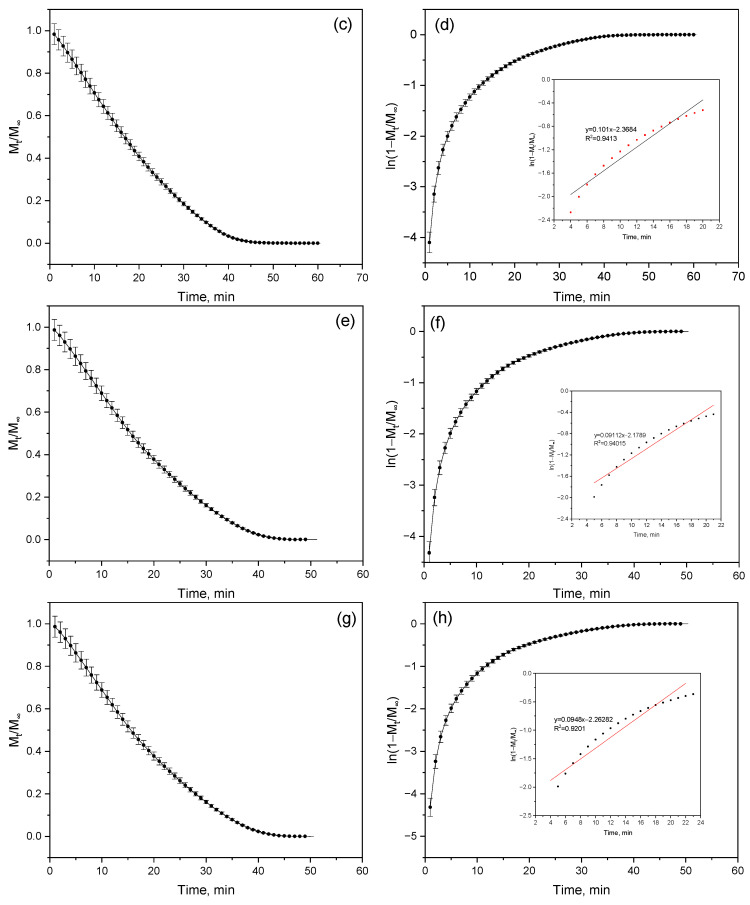
Linearized graphs of dependence $ln(1-M_{t}/M_{\infty })$ on time for PVS/PEGDA–PEGMA samples (PEGMA 11 wt.% in all samples, H_2_O 75–81 wt.%). The slope of the linear section k was used to calculate the effective diffusion coefficient D according to the Fick model. Sample compositions are as follows: (**a**,**b**)—PVA/PEGDA = 1:7 wt. ratio; (**c**,**d**)—PVA/PEGDA = 2:7 wt. ratio; (**e**,**f**)—PVA/PEGDA = 3:5 wt. ratio; (**g**,**h**)—PVA/PEGDA = 3:11 wt. ratio.

The highest slope value (k = 0.12 ± 0.001 min^−1^) was recorded for sample (a) (Figure 11a,b), corresponding to the maximum diffusion coefficient D = (2.74 ± 0.02) × 10^−8^ m^2^/min—that is, in this sample, moisture migrates within the structure at the highest rate (Table 6). A decrease in the slope for samples (b) (k = 0.10 ± 0.002 min^−1^) and (c) (k = 0.09 ± 0.002 min^−1^) leads to the expected reduction in D to (2.28 ± 0.05) × 10^−8^ m^2^/min (Figure 11c,d) and (2.05 ± 0.05) × 10^−8^ m^2^/min (Figure 11e,f), respectively. Interestingly, samples (c) and (d) exhibit identical slope values; however, their diffusion coefficients differ slightly—(2.05 ± 0.05) × 10^−8^ and (2.16 ± 0.23) × 10^−8^ m^2^/min, respectively. Although the difference is small, it indicates the influence of microstructural factors—specifically, the distribution of pores and capillaries within the polymer. Such variations affect the initial moisture content of the samples, as in more porous materials, moisture is retained differently, altering the efficiency of its transport.

A decrease in the coefficient of determination (R^2^ = 0.92) for sample (d) suggests a deviation from a purely diffusion-controlled mechanism (Figure 11g,h), indicating that moisture transfer in this case does not fully obey Fick’s law and may involve additional processes such as sorption or structural relaxation.

Thus, a clear trend is observed: as the dehydration rate (slope *k*) decreases, the diffusion coefficient also decreases. The slower the water evaporates, the more difficult it becomes for it to diffuse through the polymer matrix. This reflects increased resistance to moisture transfer due to structural densification, which reduces water permeability and enhances the material’s stability under humid conditions.

Analysis of thermogravimetric data and dehydration kinetics showed that the high dehydration rate of sample (a) is consistent with its simplified kinetics and moderate stability, as determined from 3D graphs (Figure 7 and Figure 8). For sample (b), lower diffusion coefficient values reflect an energetic but less stable process, which is confirmed by its high reactivity on thermal maps (Figure 10) and reduced $\overset{-}{A}$ values (Table 3). Sample (c) is characterized by minimal diffusion coefficients and a narrow dehydration window, which coincides with the parameters of the Šesták–Berggren model (Table 3), indicating strong diffusion limitations. Sample (d), similar to the thermolysis results, demonstrates intermediate behavior: moderate diffusion coefficient values (Table 5) and an extended temperature range of activity on 3D graphs.

Thus, the combination of thermogravimetric analysis, approximation, and calculations based on the Fick model proves that changes in composition control both the destruction mechanism and moisture transfer.

**Figure 12:**
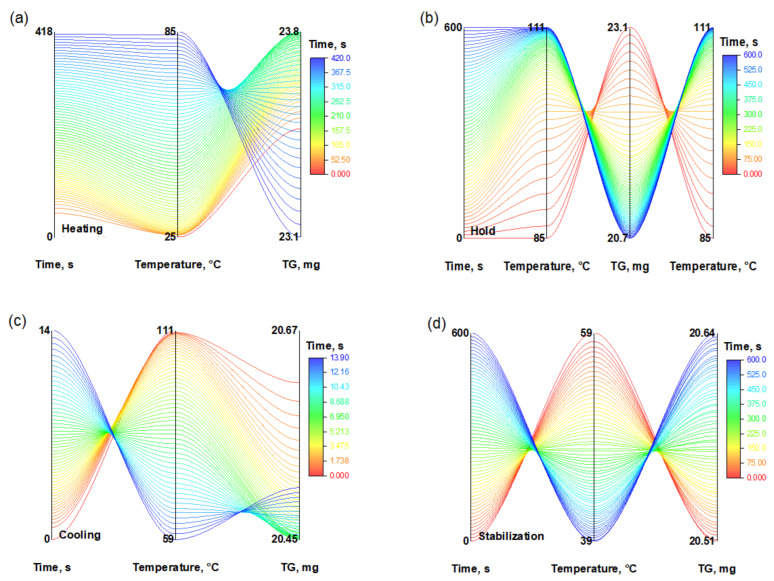
Evolution of the sample mass of PVA/PEGDA 3/11 (TG, mg), temperature, and time during a single complete thermal cycle consisting of four stages: (**a**) heating from 30 to 100 °C at a rate of 10 °C/min; (**b**) holding at 100 °C for 10 min; (**c**) cooling to 30 °C at a rate of 5 °C/min; and (**d**) stabilization at 30 °C for 10 min.

In addition to kinetic and diffusion analysis, the thermal stability of the PVA/PEGDA (3/11) hydrogel under repeated thermal exposure was evaluated. Thermogravimetric analysis (TG) was used to monitor mass changes during 20 consecutive thermal cycles, each consisting of heating (up to 100 °C), isothermal holding, cooling, and subsequent stabilization at 30 °C. As shown in Figure 12, each stage is characterized by a specific temperature profile and corresponding mass variations.

The most pronounced mass losses (Figure 12) were recorded during the isothermal holding stage, which can be attributed to thermal decomposition and desorption processes. In contrast, during the cooling and stabilization stages, slower TG changes or slight mass recovery were observed, likely due to moisture reabsorption.

The color scale represents the duration of the experiment. The graphs illustrate the decrease in sample mass at each stage, reflecting the degradation and evaporation processes occurring during the thermal cycle.

**Figure 13:**
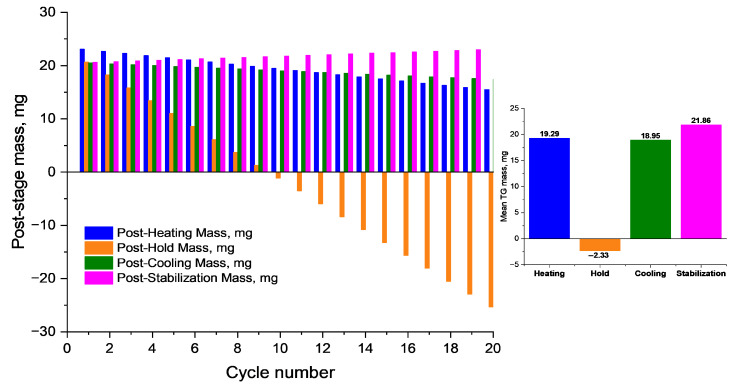
Change in sample mass of PVA/PEGDA 3/11 (TG, mg) at different stages of the thermal cycle over 20 consecutive cycles.

The summarized data (Figure 13) show that after 20 cycles, the sample retains more than 65% of its initial mass, confirming its thermal stability and reversible moisture exchange capability—key characteristics for use in biomedical devices, drug delivery systems, and smart materials operating under cyclic thermal conditions.

The results obtained in the present study expand and complement previously published data on the thermal stability of PVA- and PEG-based hydrogels [9,10,17,18]. In contrast to the aforementioned studies, which focused either on physically or chemically crosslinked systems separately, or employed limited kinetic analysis methods, our work is based on the investigation of interpenetrating polymer networks (IPNs) incorporating both PEGDA and PEGMA. It also implements a comprehensive approach that combines several modeling and statistical methods (Friedman, OFW, NPK, Šesták–Berggren, ANOVA, and *t*-tests). This approach enabled a more detailed characterization of the stages of thermal degradation, their kinetic parameters, and their dependence on composition, thereby expanding the possibilities for controlling the thermal stability of hydrogels in applied contexts, including biomedical and engineering applications.

## 4. Conclusions

A comprehensive study of the thermal behavior and kinetics of IPN hydrogels PVA/PEGDA–PEGMA (PEGMA 11 wt.% in all samples, H_2_O 75–81 wt.%) was conducted using TGA/DTG, non-model, non-parametric, and model approaches, which allowed for a comprehensive characterization of their destructive processes. It was found that an increase in PVA content enhances moisture retention and shifts the T_max_ of dehydration to higher temperatures, while an increase in PEGDA concentration leads to network densification, reduced moisture binding, and accelerated decomposition. The kinetic analysis showed differences in the behavior of the studied samples: sample (a) is characterized by moderate reactivity and a simple kinetic model; sample (b) exhibits the maximum activity at low activation energy; sample (c) is characterized by strong diffusion limitations and a narrow activity window; sample (d) demonstrates a prolonged destruction mechanism with preservation of activity in the later stages. The results obtained are of practical importance, as they allow for the targeted selection of hydrogel compositions for specific applications, including medicine (drug delivery systems, temporary implants), ecology (sorbents, biodegradable materials), and materials science (heat-resistant coatings, barrier membranes, and long-acting biomaterials).

Thus, a clear trend can be observed: as the dehydration rate (slope *k*) decreases, the diffusion coefficient D decreases as well, reflecting an increase in resistance to moisture mass transfer within the polymer matrix.

## Figures and Tables

**Table 1:** Thermal characteristics of PVA/PEGDA–PEGMA samples (PEGMA 11 wt.% in all samples, H_2_O 75–81 wt.%): (a) 1/7; (b) 2/7; (c) 3/7; (d) 3/11 (PVA/PEGDA wt.%).

Sample	Δm of Dehydration (%)	T_max_ of Dehydration (°C)	Main Destruction Interval (°C)	T_max_ of Destruction (°C)	FWHM (°C)	Residual Mass (%)
(a) PVA/PEGDA 1/7	77.8	140.0	376.0–492.0	445.0	68.1	0.4
(b) PVA/PEGDA 2/7	75.0	187.7	376.0–488.0	443.0	59.6	0.5
(c) PVA/PEGDA 3/7	83.9	135.0	383.0–487.0	453.0	80.3	0.6
(d) PVA/PEGDA 3/11	57.7	137.5	240.0–510.0	455.0	87.3	0.3

**Table 2:** Activation energy (*E_a_*, kJ mol^−1^) as a function of conversion (α), calculated using the Friedman and Ozawa–Flynn–Wall methods for the compounds studied.

Sample/α	Method	0.1	0.3	0.5	0.7	0.9
PVA/PEGDA 1/7	Friedman	101.87 ± 0.15	95.09 ± 0.20	92.66 ± 0.15	87.73 ± 0.20	86.86 ± 0.15
	OFW	98.82 ± 0.15	92.24 ± 0.20	89.88 ± 0.15	85.10 ± 0.20	84.26 ± 0.15
PVA/PEGDA 2/7	Friedman	107.47 ± 0.25	95.54 ± 0.25	93.03 ± 0.15	86.81 ± 0.20	86.78 ± 0.15
	OFW	104.25 ± 0.25	92.67 ± 0.25	90.24 ± 0.15	84.21 ± 0.20	84.18 ± 0.15
PVA/PEGDA 3/7	Friedman	102.96 ± 0.21	95.81 ± 0.10	93.24 ± 0.15	89.44 ± 0.25	84.49 ± 0.20
	OFW	99.87 ± 0.21	92.94 ± 0.10	90.44 ± 0.15	86.76 ± 0.25	81.96 ± 0.20
PVA/PEGDA 3/11	Friedman	102.67 ± 0.15	98.74 ± 0.25	93.07 ± 0.15	87.95 ± 0.15	87.23 ± 0.25
	OFW	99.59 ± 0.15	95.78 ± 0.25	95.78 ± 0.25	85.31 ± 0.15	84.61 ± 0.25

Note: All values are given as mean ± standard deviation (*n* = 3).

**Table 3:** Statistical analysis of activation energy (*E_a_*) values for hydrogel samples calculated using the Friedman and Ozawa-Flynn-Wall methods.

Composition	F-Statistic	*p*-Value (ANOVA)	t-Statistic (*t*-Test)	*p*-Value (*t*-Test)
PVA/PEGDA 1/7	0.54	0.48	34.16	0.00
PVA/PEGDA 2/7	0.28	0.61	24.69	0.00
PVA/PEGDA 3/7	0.42	0.54	29.77	0.00
PVA/PEGDA 3/11	0.16	0.70	1.55	0.20

**Table 4:** Calculated values of activation energy and pre-exponential factor for thermal decomposition of IPN gels PVA/PEGDA–PEGMA (PEGMA 11 wt.% in all samples, H_2_O 75–81 wt.%).

Sample	${\bar{E}}_{NPK}$, kJ mol^−1^ ±SD	$\overset{-}{A}$ · 10^13^, s^−1^ ±SD	Šesták–Berggren		${\bar{E}}_{S-B}$, kJ mol^−1^ ±SD	$\overset{-}{A}$ · 10^13^, s^−1^ ±SD
			*α^m^*(1 − *α*)*^n^*			
			*m*	*n*		
(a) PVA/PEGDA 1/7	102.70 ± 0.38	29.30 ± 0.20	0.64	1.15	102.31 ± 0.20	22.50 ± 0.20
(b) PVA/PEGDA 2/7	93.67 ± 0.45	1.45 ± 0.05	0.62	1.05	93.68 ± 0.15	1.59 ± 0.04
(c) PVA/PEGDA 3/7	92.87 ± 0.35	0.05 ± 0.01	0.48	0.45	93.61 ± 0.20	0.12 ± 0.01
(d) PVA/PEGDA 3/11	103.59 ± 0.26	0.04 ± 0.01	0.62	1.05	103.49 ± 0.20	0.19 ± 0.01

Note: Activation energy (*E_a_*) is given in kJ·mol^−1^; pre-exponential factor (*A*) is given in s^−1^. All values are presented as the mean ± standard deviation based on the results of three independent measurements.

**Table 5:** Statistical analysis of activation energy (*Ea*) values for IPN hydrogel samples of PVA/PEGDA–PEGMA (PEGMA 11 wt.% in all samples, H_2_O 75–81 wt.%), calculated using the NPK and Šesták–Berggren methods.

Composition	F-Statistic	*p*-Value (ANOVA)	t-Statistic (*t*-Test)	*p*-Value (*t*-Test)
(a) PVA/PEGDA 1/7	0.54	0.48	34.16	0.00
(b) PVA/PEGDA 2/7	0.28	0.61	24.69	0.00
(c) PVA/PEGDA 3/7	0.42	0.54	29.77	0.00
(d) PVA/PEGDA 3/11	0.16	0.70	1.55	0.20

**Table 6:** Kinetic parameters of dehydration and diffusion coefficients obtained using the Fick model. All values are presented as the mean ± standard deviation calculated from the results of three independent experiments (*n* = 3).

Sample	Slope *k* (min^−1^)	R^2^ Approximations	Thickness 3L (mm)	D·10^−8^, m^2^/min	D·10^−10^, m^2^/s
(a) PVA/PEGDA 1/7	0.12 ± 0.001	0.94	3	2.74 ± 0.02	4.56 ± 0.04
(b) PVA/PEGDA 2/7	0.10 ± 0.002	0.94	3	2.28 ± 0.05	3.80 ± 0.08
(c) PVA/PEGDA 3/7	0.09 ± 0.002	0.94	3	2.05 ± 0.05	3.42 ± 0.08
(d) PVA/PEGDA 3/11	0.09 ± 0.01	0.92	3	2.16 ± 0.23	3.60 ± 0.38

## Data Availability

The original contributions presented in this study are included in the article. Further inquiries can be directed to the corresponding author.

## Supplementary Materials

The following supporting information can be downloaded at: https://www.mdpi.com/article/10.3390/polym17202805/s1, Supplementary Materials S1: Rheological Behavior of PVA/PEGDA–PEGMA Hydrogels with Varying PVA Content; Figure S1: Frequency dependencies of accumulation G′ (filled symbols) and G″ (unfilled symbols) for hydrogels with different PVA content after a freeze cycle. Fixed PEGMA content of 11 wt.%. Temperature: 20 °C. Supplementary Materials S2: Macroscopic and SEM Morphology of PVA/PEGDA 3/7 Hydrogels; Figure S2: Morphological characterization of the PVA/PEGDA–PEGMA 3/7 hydrogel (PEGMA 11 wt.% in all samples, H2O 78 wt.%): (a) macrophotograph of the hydrogel after freeze–thaw processing, demonstrating uniform structure and stable shape; (b) SEM image at 1000× magnification, showing porous morphology indicative of microphase separation within the polymer network. Supplementary Materials S3: Rheological, morphological, and structural characteristics of hydrogels; Figure S3: Frequency dependences of the storage G′ (filled symbols) and G″ (open symbols) for PEGMA/PEGDA (squares); PEGMA/PEGDA/PVA hydrogel (circles) and PEGMA/PEGDA/PVA after freezing (triangles) hydrogels. Temperature: 20 °C; Figure S4: Frequency dependencies of the storage modulus (G′) for PEGMA/PEGDA11/PVA3 after freezing hydrogels with different content of PEGMA (a) and PEGMA11/PEGDA/PVA3 after freezing hydrogels with different content of PEGDA (b). Temperature: 20 °C; Figure S5: Dependencies of the elastic modulus G_0_ on PVA concentration in PEGMA11/PEGDA5/PVA (triangles), PEGMA11/PEGDA5/PVA after freezing (circles) and PEGMA11/PEGDA5/PVA after freezing and tannic acid (squares) hydrogels. Temperature: 20 °C. The lines connecting experimental points serve to guide the eye; Figure S6: X-ray diffraction patterns of the PEGMA/PEGDA/PVA (black) and PEGMA/PEGDA/PVA after freezing (blue) Temperature: 20 °C. Arrow points out an additional XRD signal after freezing and thawing of the gel; Figure S7: ATP FTIR spectra of PEGMA/PEGDA hydrogel (black); PEGMA/PEGDA/PVA hydrogel (orange); PEGMA/PEGDA/PVA after freezing (red) and PEGMA/PEGDA/PVA after freezing and tannic acid (blue). Supplementary Materials S4: IR Spectral Analysis of PVA/PEGDA–PEGMA Hydrogels at Different Heating Temperatures; Figure S8: IR spectra of compositions based on PVA/PEGDA–PEGMA (PEGMA 11 wt.% in all samples, H2O 75–81 wt.%) at different heating temperatures: (a)—composition with a PVA/PEGDA ratio of 1:7; (b)—composition with a PVA/PEGDA ratio of 2:7; Supplementary Materials S5: Thermal Evolution of the Morphology of PVA/PEGDA–PEGMA Hydrogels by SEM; Figure S9: SEM images of the PVA/PEGDA–PEGMA composition (PEGMA 11 wt.% in all samples, H_2_O 75–81 wt.%, PVA/PEGDA = 2:7) after heat treatment at 150 °C: (a) ×100,000—relatively dense surface with nanocracks and small inclusions, reflecting the onset of dehydration and defect formation; (b) ×5000—smooth areas with a network of cracks caused by thermal stresses; (c) ×1000—morphology with folds and microcracks indicating surface shrinkage; (d) ×200—the film remains intact, but a network of cracks is visible, indicating the initial stage of degradation. These morphological features correspond to the early stage of thermal exposure, when the structure still retains its density, but defects are already forming, preceding pore formation at higher temperatures; Figure S10: SEM images of the PVA/PEGDA–PEGMA composition (PEGMA 11 wt.% in all samples, H_2_O 75–81 wt.%, PVA/PEGDA = 2:7) after heat treatment at 250 °C: (a) ×50,000—nanopores with a diameter of 0.5–2 μm with thin partitions formed as a result of initial gas release; (b) ×10,000—uniformly distributed spherical pores (1–5 μm) on a relatively smooth surface, reflecting the onset of swelling; (c) ×1000—clusters of pores of various sizes (from single digits to tens of microns), indicating their coalescence and growth; (d) ×200—the film remains intact, but wavy deformations caused by internal stresses are visible. These morphological features indicate an early stage of destruction: the formation of bubbles and porosity while maintaining the overall structure of the matrix; Figure S11: SEM images of the PVA/PEGDA–PEGMA composition (PEGMA 11 wt.% in all samples, H_2_O 75–81 wt.%, PVA/PEGDA = 2:7) after heat treatment at 450 °C: (a) ×50,000—surface with nanopores (50–130 nm) formed due to gas release; (b) ×5000—relatively smooth surface with small inclusions, indicating partial preservation of the polymer matrix; (c) ×500—wave-like folds and cracks caused by shrinkage and internal stresses; (d) ×200—macro-breaks and crater-like cavities, indicating intense gas release and the onset of carbonization. The multi-level morphology indicates a transitional stage of destruction: the formation of pores and loss of structural integrity without complete carbonization (unlike at 600 °C); Figure S12: SEM images of the PVA/PEGDA–PEGMA composition (PEGMA 11 wt.% in all samples, H_2_O 75–81 wt.%, PVA/PEGDA = 2:7) after heat treatment at 600 °C: (a) crystallite-like domains (0.5–1.2 μm) with dense packing; (b) porous carbon structure with cellular pores and channels (0.9–4.4 μm) formed as a result of gas evolution; (c) large flake-like fragments (50–150 μm) with folded and layered morphology; (d) carbonized residues on the substrate with craters formed by the release of volatile products (50–300 μm). These multi-level morphological features reflect intense gas release, local heat accumulation, and partial carbonization of the polymer matrix. Supplementary Materials S6: Kinetic Model Complementarity; Table S1: Comparison of the contributions of kinetic models.

## Abbreviations

The following abbreviations are used in this manuscript:

PEGMA	Poly(ethyleneglycol) methacrylate
PEGDA	Poly(ethyleneglycol) diacrylate
PVA	poly(vinyl alcohol)
TPO-Li	Lithium 2,4,6-trimethylbenzoyl phosphinate
LM	light microscopy
cryo-LVSEM	cryogenic low-vacuum scanning electron microscopy
cryo-SEM	cryogenic scanning electron microscopy
AA	PVA «Alfa Aesar, MW 41000, 98–99%»
OFW	Ozawa–Flynn–Wall
