# Supplementary material for: Thermal Stability and Decomposition Mechanisms of PVA/PEGDA–PEGMA IPN-Hydrogels: A Multimethod Kinetic Approach
Source: Polymers (Basel). 2025 Oct 21;17(20):2805. doi: 10.3390/polym17202805 (PMC12566940; doi:10.3390/polym17202805)
Supplement: Supplementary file 1 [file polymers-17-02805-s001.zip › Supplementary Materials S1.pdf]

Supplementary Materials S1

Rheological Behavior of PVA/PEGMA/PEGDA Hydrogels with Varying PVA Content

Rheological measurements were performed using an Anton Paar MCR302 rheometer (Anton Paar, Austria) with a controlled shear stress and a plate-plate measuring cell (lower plate P-LP25/AL/G1; upper plate PP25). The gap between the plates was 5 mm. The dependence of the storage modulus G' and loss modulus G'' on the applied stress frequency ω was measured in the frequency range 0.05–500 rad/s at a deformation amplitude of 0.1%, which corresponds to the linear viscoelastic region of hydrogels. The temperature was maintained at 20 ± 0.5 °C using Peltier elements.

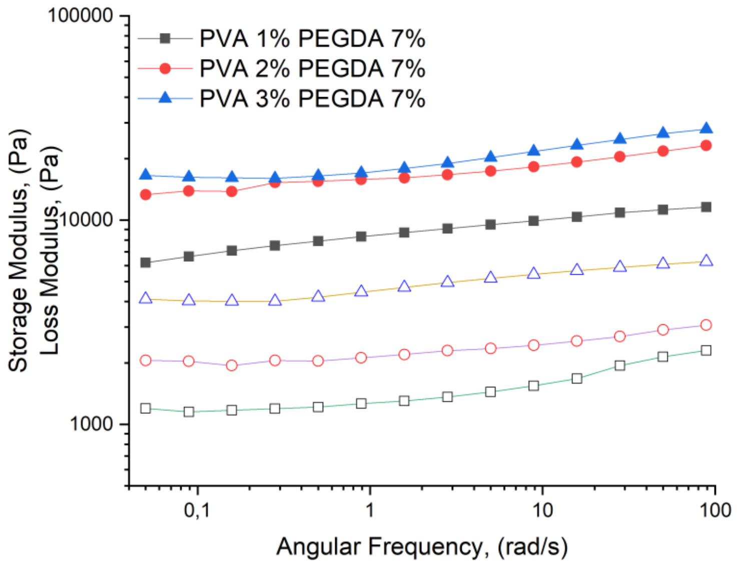

Figure S1. Frequency dependencies of accumulation G' (filled symbols) and G'' (unfilled symbols) for hydrogels with different PVA content after a freeze cycle. Fixed PEGMA content of 11 wt.%. Temperature: 20°C

Figure S1 shows the dependence of the storage modulus (G') and loss modulus (G'') on angular frequency for all compositions. In all cases, the values of G' significantly exceed those of G'' across the entire frequency range, indicating predominantly elastic behavior characteristic of cross-linked hydrogels. An increase in PVA concentration from 1% to 3% leads to a significant increase in G', reflecting an increase in the elasticity and stiffness of the gel network. The increase in G'' is less pronounced, which further confirms the dominance of the elastic component over the viscous one. The weak frequency dependence of both modules, especially at low PVA concentrations, indicates the formation of a stable and developed three-dimensional network. The results obtained suggest that PEGDA and PEGMA, activated by a photoinitiator, form a three-dimensional network structure, while

PVA forms an additional physical network due to hydrogen bonds and microcrystals formed during freezing at –25°C and subsequent thawing at room temperature, which strengthens the material and determines the values of the modules.
