# Supplementary material for: Thermal Stability and Decomposition Mechanisms of PVA/PEGDA–PEGMA IPN-Hydrogels: A Multimethod Kinetic Approach
Source: Polymers (Basel). 2025 Oct 21;17(20):2805. doi: 10.3390/polym17202805 (PMC12566940; doi:10.3390/polym17202805)
Supplement: Supplementary file 1 [file polymers-17-02805-s001.zip › Supplementary Materials S2.pdf]

Supplementary Materials S2

Macroscopic and SEM Morphology of PVA/PEGDA 3/7 Hydrogels

Sample PVA/PEGDA 3/7 was chosen for morphological characterization, as it exhibited the lowest activation energy and distinctive kinetic behavior. The macrophotograph and SEM image illustrate its porous morphology and phase heterogeneity.

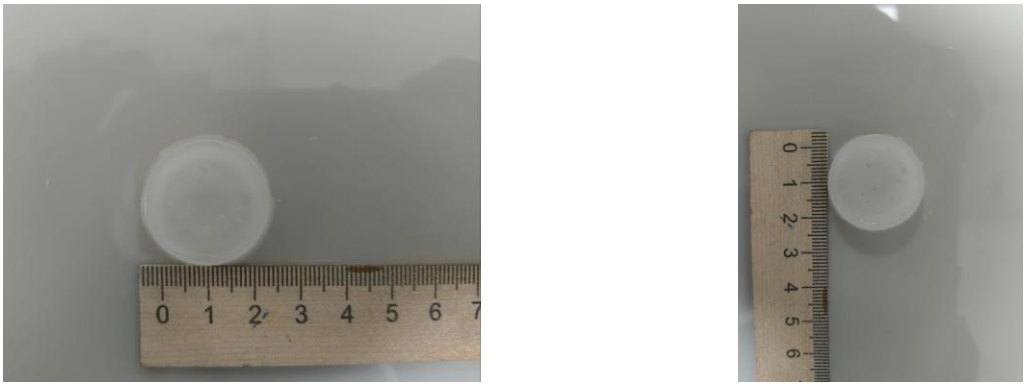

(a)

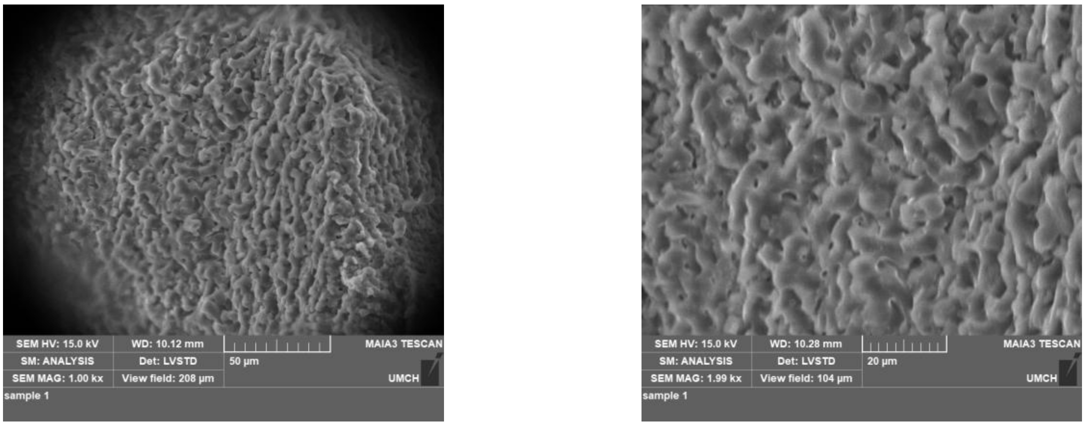

(b)

Figure S2. Morphological characterization of the PVA/PEGDA–PEGMA 3/7 hydrogel (PEGMA 11 wt.% in all samples, H_2O 78 wt.%): (a) macrophotograph of the hydrogel after freeze–thaw processing, demonstrating uniform structure and stable shape; (b) SEM image at 1000× magnification, showing porous morphology indicative of microphase separation within the polymer network
