# Supplementary material for: Thermal Stability and Decomposition Mechanisms of PVA/PEGDA–PEGMA IPN-Hydrogels: A Multimethod Kinetic Approach
Source: Polymers (Basel). 2025 Oct 21;17(20):2805. doi: 10.3390/polym17202805 (PMC12566940; doi:10.3390/polym17202805)
Supplement: Supplementary file 1 [file polymers-17-02805-s001.zip › Supplementary Materials S3.pdf]

Supplementary Materials S3

Rheological, Morphological, and Structural Characteristics of Hydrogels

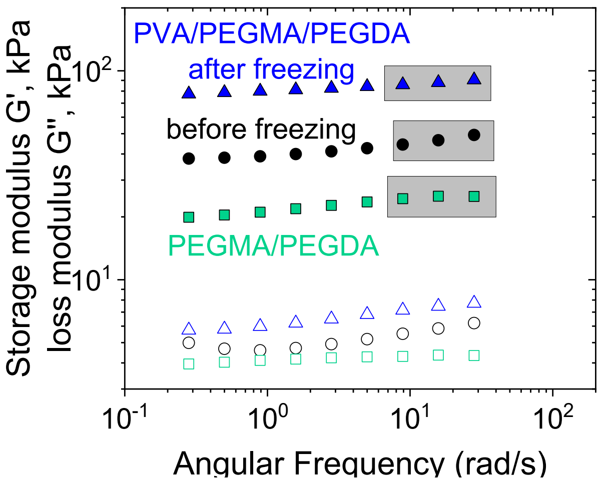

Figure S3. Frequency dependences of the storage G′ (filled symbols) and G″ (open symbols) for PEGMA /PEGDA (squares); PEGMA/PEGDA/PVA hydrogel (circles) and PEGMA/PEGDA/PVA after freezing (triangles) hydrogels. Temperature: 20°C.

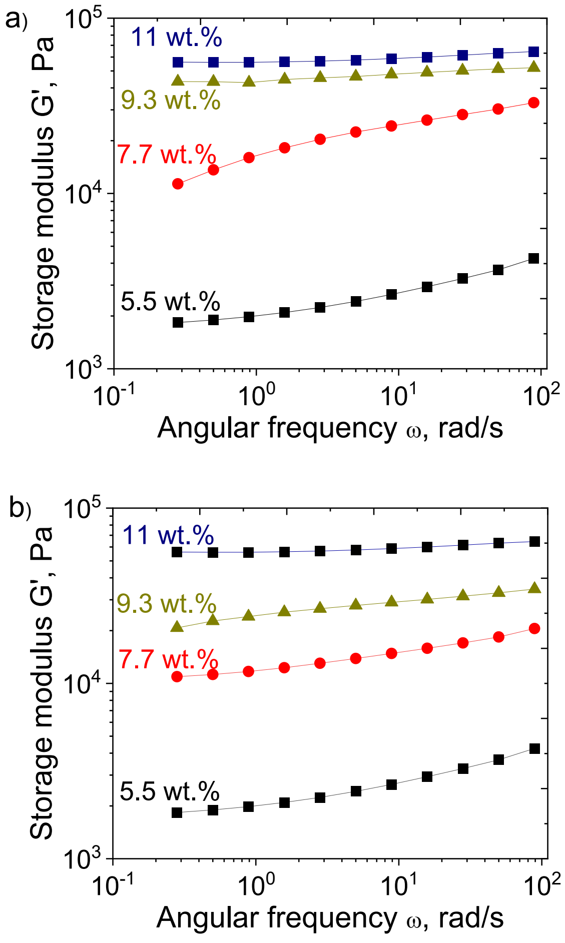

Figure S4. Frequency dependencies of the storage modulus (G′) for PEGMA/PEGDA11/PVA3 after freezing hydrogels with different content of PEGMA (a) and PEGMA11/PEGDA/PVA3 after freezing hydrogels with different content of PEGDA (b). Temperature: 20°C.

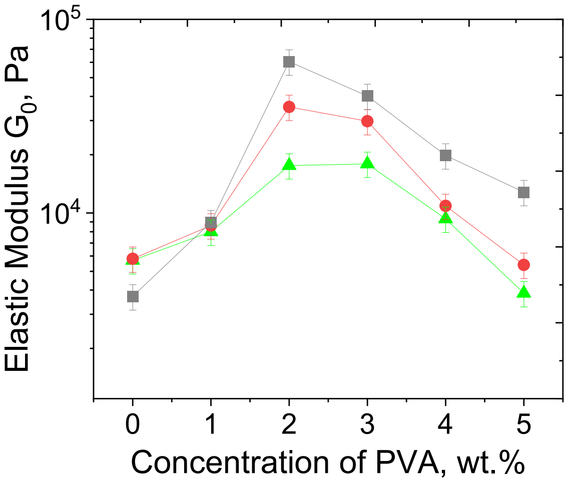

Figure S5. Dependencies of the elastic modulus G_0 on PVA concentration in PEGMA11/PEGDA5/PVA (triangles), PEGMA11/PEGDA5/PVA after freezing (circles) and PEGMA11/PEGDA5/PVA after freezing and tannic acid (squares) hydrogels. Temperature: 20°C. The lines connecting experimental points serve to guide the eye.

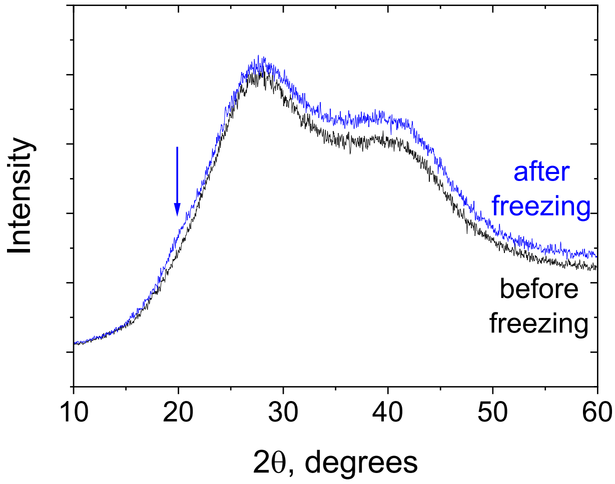

Figure S6. X-ray diffraction patterns of the PEGMA/PEGDA/PVA (black) and PEGMA/PEGDA/PVA after freezing (blue) Temperature: 20°C. Arrow points out an additional XRD signal after freezing and thawing of the gel.

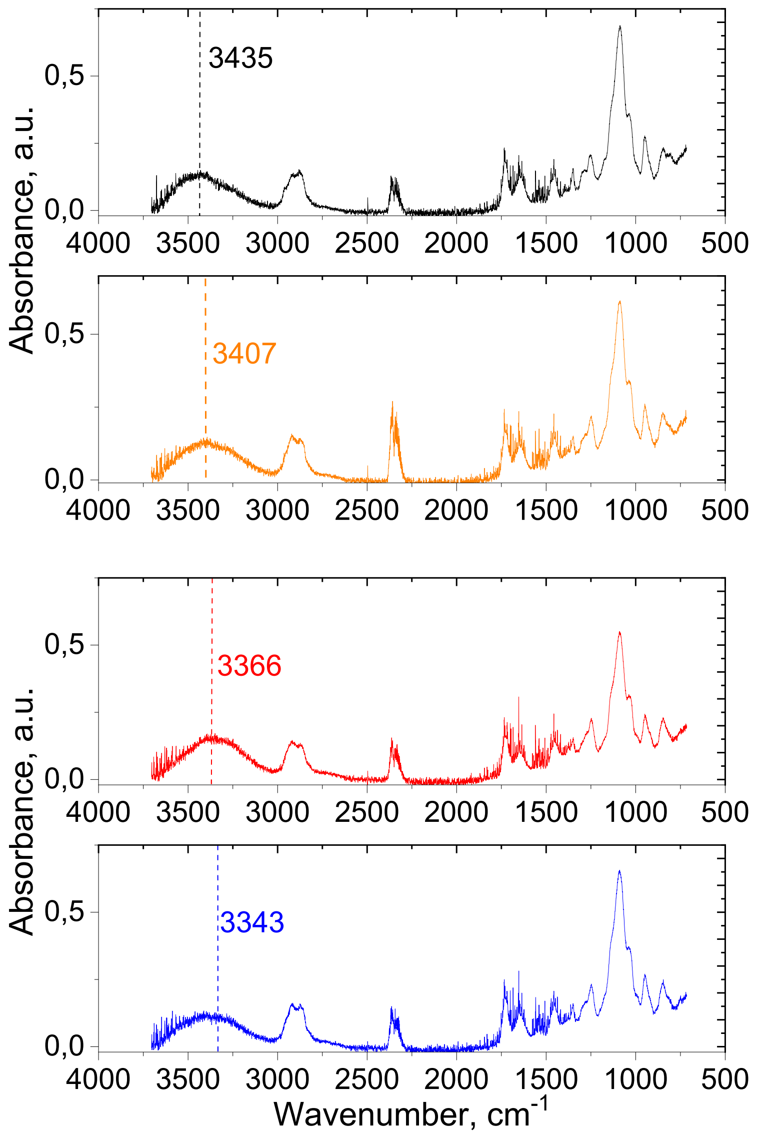

Figure S7. ATP FTIR spectra of PEGMA/PEGDA hydrogel (black); PEGMA/PEGDA/PVA hydrogel (orange); PEGMA/PEGDA/PVA after freezing (red) and PEGMA/PEGDA/PVA after freezing and tannic acid (blue).
