# Supplementary material for: Thermal Stability and Decomposition Mechanisms of PVA/PEGDA–PEGMA IPN-Hydrogels: A Multimethod Kinetic Approach
Source: Polymers (Basel). 2025 Oct 21;17(20):2805. doi: 10.3390/polym17202805 (PMC12566940; doi:10.3390/polym17202805)
Supplement: Supplementary file 1 [file polymers-17-02805-s001.zip › Supplementary Materials S4.pdf]

Supplementary Materials S4

IR Spectral Analysis of PVA/PEGDA–PEGMA Hydrogels at Different Heating Temperatures

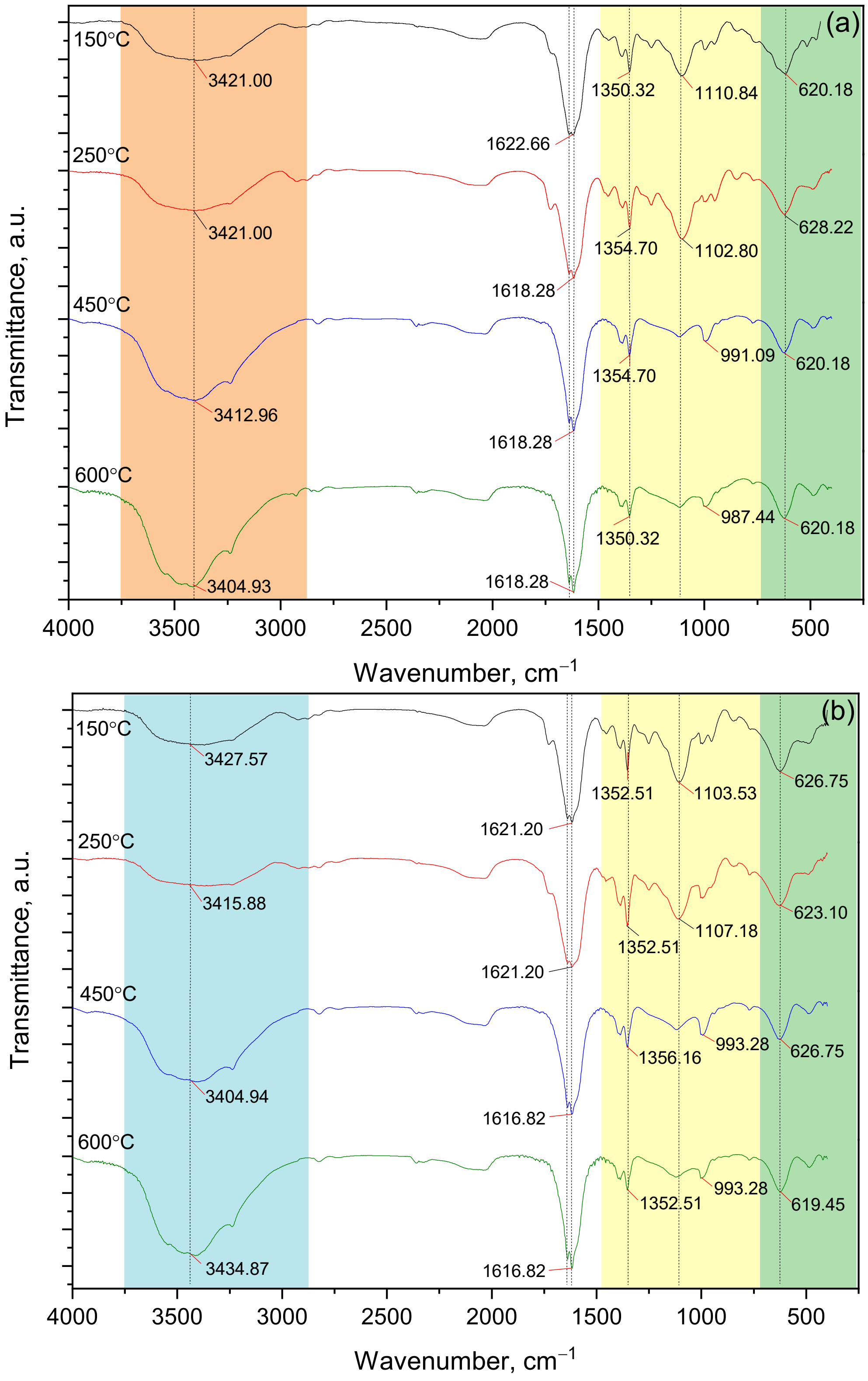

Figure S8. IR spectra of compositions based on PVA/PEGDA–PEGMA (PEGMA 11 wt.% in all samples, H_2O 75–81 wt.%) at different heating temperatures: (a) — composition with a PVA/PEGDA ratio of 1:7; (b) — composition with a PVA/PEGDA ratio of 2:7

Wave number (cm^{-1})	The nature of vibrations	The nature of vibrations
(a) PVA/PEGDA 1/7		
3421.00–3404.93	–OH	Water, –OH PVA; when heated → increase in width and intensity, indicating moisture adsorption and the appearance of new –OH on the residues
1622.66–1618.28	C=C (aromatic/conjugate systems)	At low PVA content, the structure breaks down faster → carbon fragments and aromatic rings form more actively. An increase in the –OH peak at 600°C confirms carbonization and hygroscopicity of the residue.
1354.70–1350.32	$\delta(CH_2)$	Methylene groups of PVA; peak remains, shifts slightly
1110.84–1102.80	ν(C–O–C)	PEGDA/PEGMA ether bridges; peaks shift and weaken → destruction of polyester fragments
991.09–987.44	δ(–OH), –CH	Deformations –OH and –CH; intensity decreases with temperature
620.18–628.22	Skeletal vibrations	Residues from structure or thermolysis products; peaks remain, minor shifts
(b) PVA/PEGDA 3/11		
3427.57–3434.87	–OH	Unlike (a), here the –OH peaks are higher already in the initial stages → more PVA, more hydroxyl groups. When heated, the peak remains pronounced.

1621.20–1616.82	Conjugated C=O (acrylate/ester residues); partially C=C	With a higher proportion of PVA, more hydroxyl groups are retained, and polyester bridges are destroyed more slowly. This results in more products with carbonyl groups (acids, esters) that couple with C=C and give a signal in this region.
1356.16–1352.51	δ(CH_2)	Methylene bonds in PVS; the structure is more saturated with them; the peaks are more stable
1107.18–1103.53	ν(C–O–C)	Vibrations of ether groups; remain, but show less degradation than in (a)
993.28	δ(–OH), –CH	Deformation vibrations –CH and –OH; remain pronounced even at high temperatures
619.45–626.75	Skeletal	Minor shifts; more stable structure
