# Supplementary material for: Thermal Stability and Decomposition Mechanisms of PVA/PEGDA–PEGMA IPN-Hydrogels: A Multimethod Kinetic Approach
Source: Polymers (Basel). 2025 Oct 21;17(20):2805. doi: 10.3390/polym17202805 (PMC12566940; doi:10.3390/polym17202805)
Supplement: Supplementary file 1 [file polymers-17-02805-s001.zip › Supplementary Materials S5.pdf]

Supplementary Materials S5

Thermal Evolution of the Morphology of PVA/PEGDA–PEGMA Hydrogels by SEM

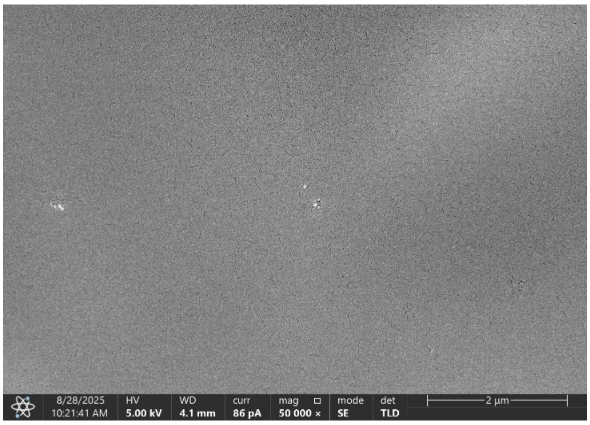

(a) ×50 000

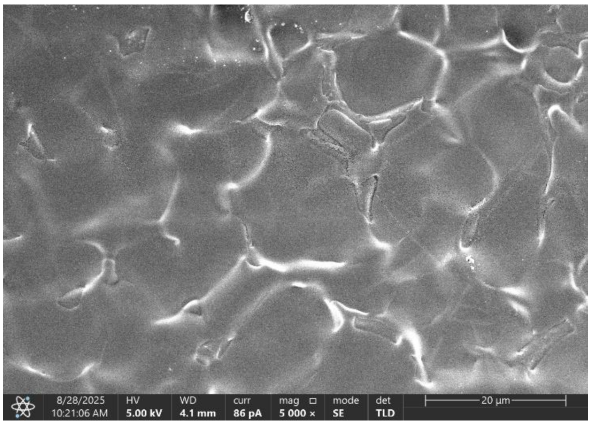

(b) ×5 000

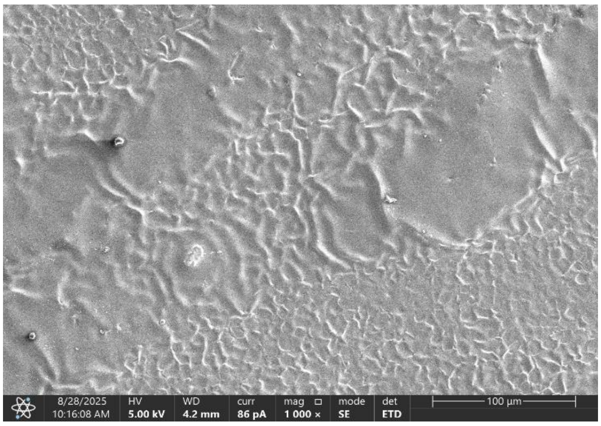

(c) ×1000

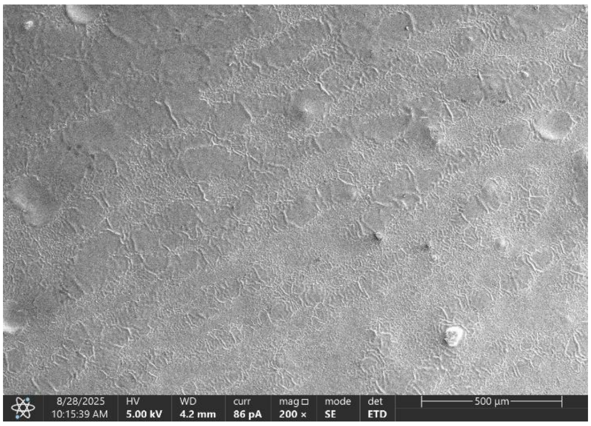

(d) ×200

Figure S9. SEM images of the PVA/PEGDA–PEGMA composition (PEGMA 11 wt.% in all samples, H_2O 75–81 wt.%, PVA/PEGDA = 2:7) after heat treatment at 150°C: (a) ×100,000 – relatively dense surface with nanocracks and small inclusions, reflecting the onset of dehydration and defect formation; (b) ×5,000 – smooth areas with a network of cracks caused by thermal stresses; (c) ×1,000 – morphology with folds and microcracks indicating surface shrinkage; (d) ×200 – the film remains intact, but a network of cracks is visible, indicating the initial stage of degradation. These morphological features correspond to the early stage of thermal exposure, when the structure still retains its density, but defects are already forming, preceding pore formation at higher temperatures.

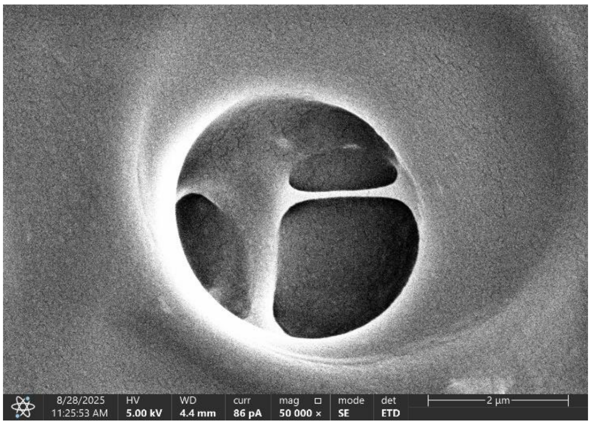

(a) ×50 000

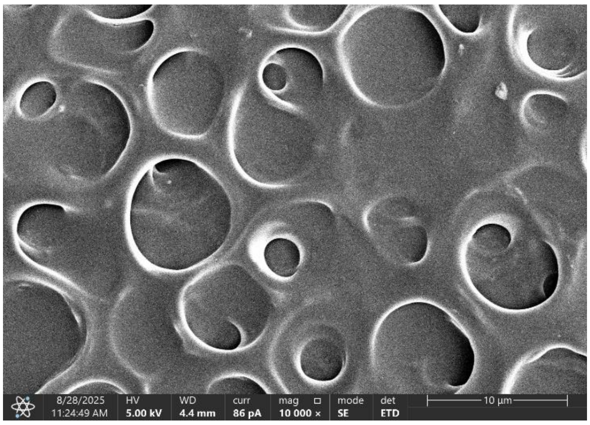

(b) ×10 000

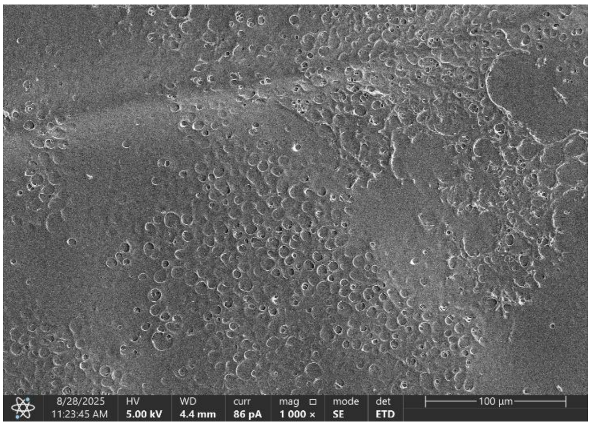

(c) ×1000

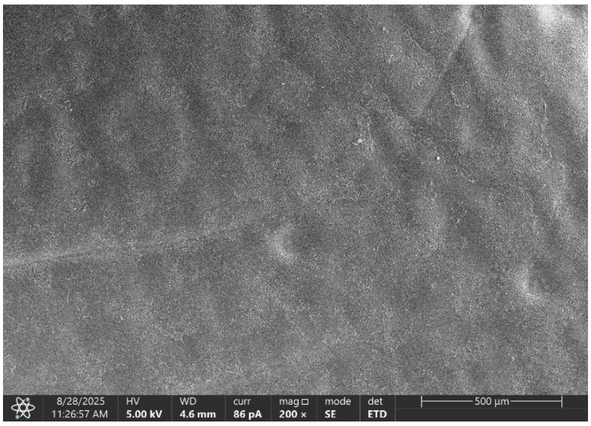

(d) ×200

Figure S10. SEM images of the PVA/PEGDA–PEGMA composition (PEGMA 11 wt.% in all samples, H_2O 75–81 wt.%, PVA/PEGDA = 2:7) after heat treatment at 250°C: (a) ×50,000 – nanopores with a diameter of 0.5–2 μm with thin partitions formed as a result of initial gas release; (b) ×10,000 – uniformly distributed spherical pores (1–5 μm) on a relatively smooth surface, reflecting the onset of swelling; (c) ×1,000 – clusters of pores of various sizes (from single digits to tens of microns), indicating their coalescence and growth; (d) ×200 – the film remains intact, but wavy deformations caused by internal stresses are visible. These morphological features indicate an early stage of destruction: the formation of bubbles and porosity while maintaining the overall structure of the matrix.

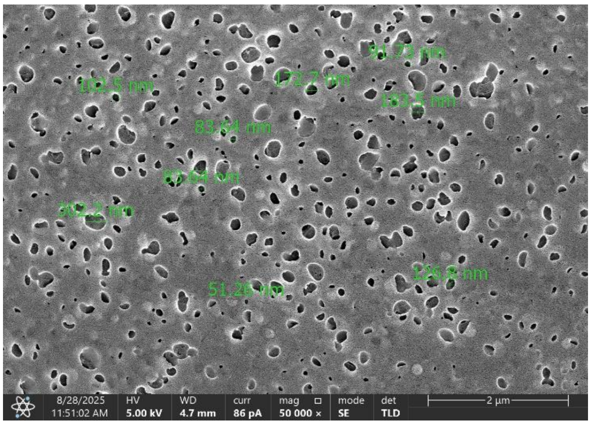

(a) ×50 000

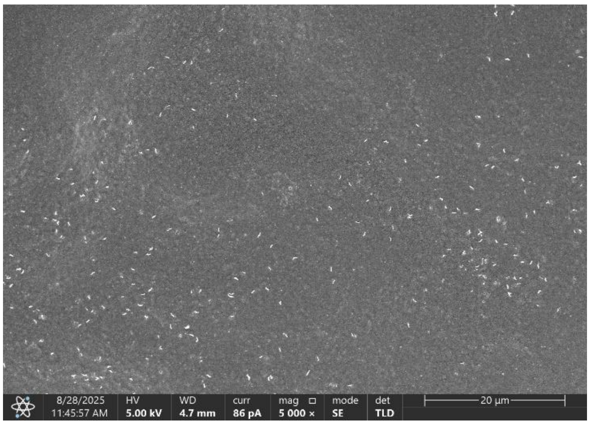

(b) ×5 000

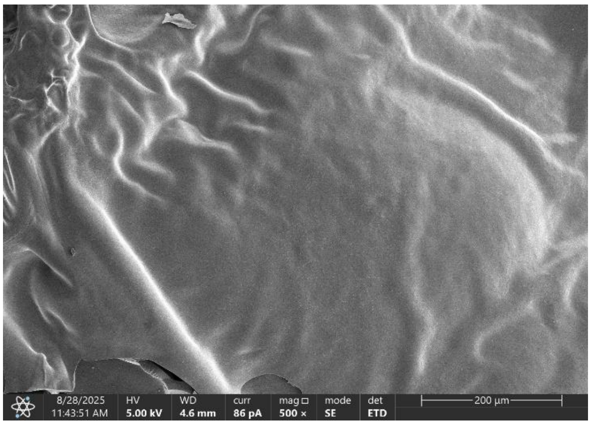

(c) ×500

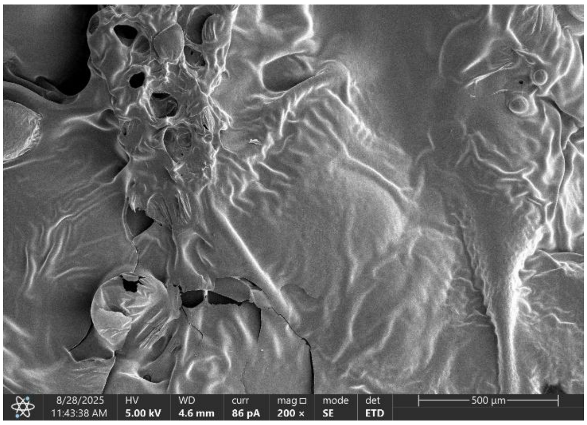

(d) ×200

Figure S11. SEM images of the PVA/PEGDA–PEGMA composition (PEGMA 11 wt.% in all samples, H_2O 75–81 wt.%, PVA/PEGDA = 2:7) after heat treatment at 450°C: (a) ×50,000 – surface with nanopores (50–130 nm) formed due to gas release; (b) ×5,000 – relatively smooth surface with small inclusions, indicating partial preservation of the polymer matrix; (c) ×500 – wave-like folds and cracks caused by shrinkage and internal stresses; (d) ×200 – macro-breaks and crater-like cavities, indicating intense gas release and the onset of carbonization. The multi-level morphology indicates a transitional stage of destruction: the formation of pores and loss of structural integrity without complete carbonization (unlike at 600°C).

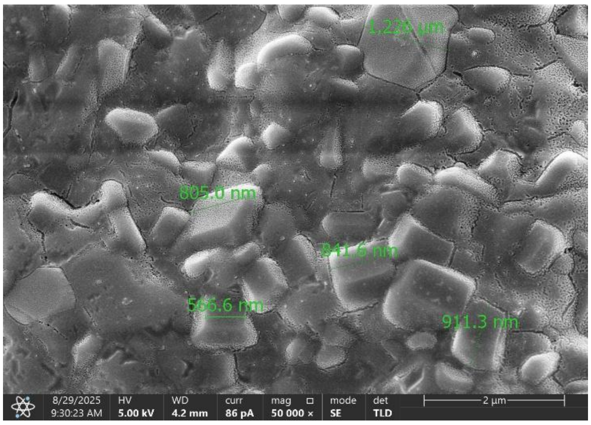

(a) ×50 000

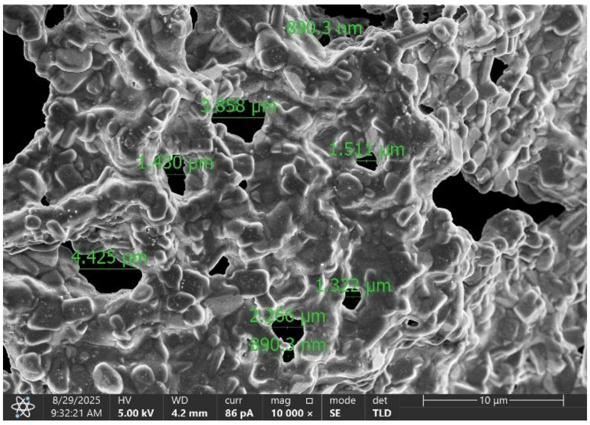

(b) ×10 000

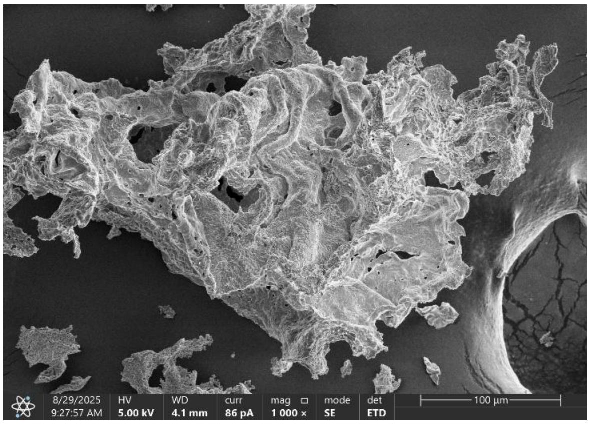

(c) ×1000

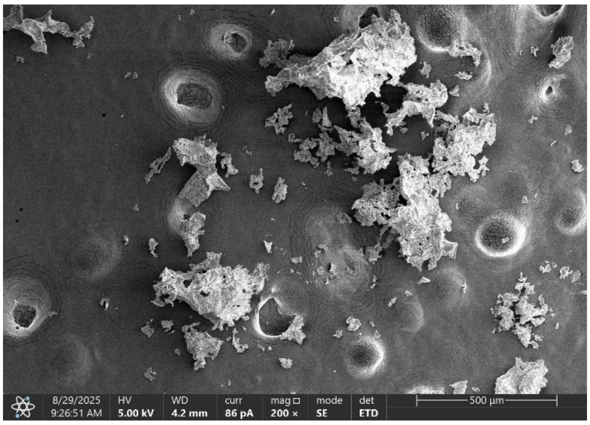

(d) ×200

Figure S12. SEM images of the PVA/PEGDA–PEGMA composition (PEGMA 11 wt.% in all samples, H_2O 75–81 wt.%, PVA/PEGDA = 2:7) after heat treatment at 600°C: (a) crystallite-like domains (0.5–1.2 μm) with dense packing; (b) porous carbon structure with cellular pores and channels (0.9–4.4 μm) formed as a result of gas evolution; (c) large flake-like fragments (50–150 μm) with folded and layered morphology; (d) carbonized residues on the substrate with craters formed by the release of volatile products (50–300 μm). These multi-level morphological features reflect intense gas release, local heat accumulation, and partial carbonization of the polymer matrix.
