# Supplementary material for: Thermal Stability and Decomposition Mechanisms of PVA/PEGDA–PEGMA IPN-Hydrogels: A Multimethod Kinetic Approach
Source: Polymers (Basel). 2025 Oct 21;17(20):2805. doi: 10.3390/polym17202805 (PMC12566940; doi:10.3390/polym17202805)
Supplement: Supplementary file 1 [file polymers-17-02805-s001.zip › Supplementary Materials S6.pdf]

Supplementary Materials S6

Kinetic Model Complementarity

The table presents the functional classification of the kinetic models applied. Their combined use made it possible not only to calculate the thermokinetic parameters, but also to obtain a more comprehensive understanding of the degradation mechanism, including the identification of its multi-stage nature, autocatalytic effects, and the nonlinear dependence of activation energy on the degree of conversion.

Table S1. Comparison of the contributions of kinetic models

Model	Type	Purpose of application	Information obtained
**Friedman**	Model-free	Calculation of E_a without assumptions about the reaction mechanism	Identified degradation stages and variations in activation energy depending on the degree of conversion (α)
**Ozawa–Flynn–Wall (OFW)**	Model-free	Independent estimation of E_a and verification of consistency	Confirmed the Friedman results; assessed the multistage nature of the process
**NPK (Non-Parametric Kinetics)**	Semi-empirical	Determination of the functional dependence $f(\alpha)$ without a predefined model	Established the shape of $f(\alpha)$ without prior assumptions; revealed complex degradation kinetics
**Šesták–Berggren**	Reaction model function	Approximation of autocatalytic behavior	Described the self-accelerated degradation typical of crosslinked structures
